# EquiDesign: a versatile computational framework for *de novo* protein sequence design

**DOI:** 10.1093/bib/bbag581

**Published:** 2026-10-26

**Authors:** Qiang Gao, Zhongyu Xu, Kai Chen, Xiaochen Cen, Ke Yan, Jinxin Liu, Zhiwei Ji

**Affiliations:** College of Artificial Intelligence, Nanjing Agricultural University, No. 666 Binjiang Avenue, Nanjing, Jiangsu 211800, China; Center for Data Science and Intelligent Computing, Nanjing Agricultural University, No. 666 Binjiang Avenue, Nanjing, Jiangsu 211800, China; College of Artificial Intelligence, Nanjing Agricultural University, No. 666 Binjiang Avenue, Nanjing, Jiangsu 211800, China; Center for Data Science and Intelligent Computing, Nanjing Agricultural University, No. 666 Binjiang Avenue, Nanjing, Jiangsu 211800, China; Research and Development-Statistics, Eli Lilly and Company, Lilly Corporate Center, Indianapolis, IN 46285, United States; College of Artificial Intelligence, Nanjing Agricultural University, No. 666 Binjiang Avenue, Nanjing, Jiangsu 211800, China; Center for Data Science and Intelligent Computing, Nanjing Agricultural University, No. 666 Binjiang Avenue, Nanjing, Jiangsu 211800, China; Department of the Built Environment, College of Design and Engineering, National University of Singapore, 4 Architecture Drive, Singapore 117566, Singapore; College of Mechanical and Vehicle Engineering, Hunan University, Changsha 410082, China; Laboratory of Gastrointestinal Microbiology, College of Animal Science and Technology, Nanjing Agricultural University, No. 1 Weigang Road, Nanjing 210095, China; College of Artificial Intelligence, Nanjing Agricultural University, No. 666 Binjiang Avenue, Nanjing, Jiangsu 211800, China; Center for Data Science and Intelligent Computing, Nanjing Agricultural University, No. 666 Binjiang Avenue, Nanjing, Jiangsu 211800, China

**Keywords:** protein design, *de novo*, residue, sequence, Equiformer

## Abstract

Designing amino acid sequences that uniquely fold into target three-dimensional conformations defines the protein inverse folding problem, a formidable bottleneck in *de novo* protein design. Current computational paradigms, including deep learning-based approaches, often struggle to reconcile high sequence accuracy with structural stability, computational efficiency, and generalizability across diverse protein families. Here, we present EquiDesign, a deep learning framework that surmounts these constraints via a novel multistep Equiformer-enhanced architecture. By disentangling geometric features into invariant scalars and equivariant vectors, EquiDesign preserves the intrinsic directional topology of the protein backbone during message passing. This is achieved through spatial K-nearest neighbor graph construction and geometric attention mechanisms that hierarchically fuse multiscale contextual information, yielding a physically grounded representation of protein conformations. Extensive benchmarking demonstrates that EquiDesign achieves superior sequence recovery on standard and challenging test sets compared with state-of-the-art methods. Critically, the generated sequences exhibit exceptional structural fidelity, folding stability, and sequence diversity, as validated by comprehensive controls against training-set memorization, compositional bias, and physical energy landscapes, alongside targeted diversity sampling. These results establish EquiDesign as a robust, scalable, and generalizable inverse folding solution, driven by SE(3)-equivariant encoding and multiscale geometric attention, thus propelling *de novo* protein design toward unprecedented structural precision.

## Introduction

Proteins are the molecular engines of life, executing a vast array of catalytic, regulatory, and structural functions dictated by their precisely folded three-dimensional (3D) conformations [1–3]. The *de novo* design of protein sequences that spontaneously fold into target native-like structures (commonly referred to as the protein inverse folding problem) remains a grand challenge in structural biology and synthetic biology, with transformative implications for therapeutics, industrial enzymes, and biosensors [4, 5]. feature extraction module is adapted

Historically, conventional computational approaches bifurcated into physics-based and statistics-based strategies. Physics-based methods, rooted in molecular mechanics, typically formulate inverse folding as a combinatorial energy minimization problem, relying on physical force fields and conformational sampling to identify stabilizing sequences for a fixed backbone [6, 7]. Despite their physical interpretability, these methods are plagued by inaccuracies in empirical energy functions and the exponential complexity of the discrete sequence space, severely limiting their scalability and robustness [8]. Conversely, statistical methods infer sequence-structure correlations from multiple sequence alignments or structural homologs [9]. While intuitive, these homology-driven approaches inherently struggle with novel folds and suffer from a critical limitation: the “data scarcity” paradox. As highlighted by recent studies, the exponential growth of sequence space vastly outstrips available annotated data, making it difficult for purely data-driven models to generalize to underrepresented or *de novo* scaffolds [10, 11].

To address these limitations, early machine learning approaches introduced a paradigm shift by leveraging pattern recognition to bypass explicit physics simulations. Methods such as support vector machines and hidden Markov models attempted to classify designability based on coarse-grained features like hydrophobic/polar patterns [12]. Neural network-based frameworks, such as SPIN, further advanced this by integrating local structural fragments with energy terms to predict compatible sequences [13]. These pioneering efforts laid the conceptual groundwork for modern deep learning, demonstrating that high-level sequence-structure relationships could be effectively captured without exhaustive sampling.

The advent of deep learning has since catalyzed a revolution in protein design, fundamentally changing how we model the relationship between sequence, structure, and function [14]. The field has rapidly evolved from static encoders to dynamic, generative architectures, with a particular emphasis on geometric deep learning​ to preserve 3D physical constraints. The introduction of models like ProteinMPNN marked a significant milestone by demonstrating that sequence recovery could be dramatically improved by operating directly on atomic coordinates and incorporating bidirectional message passing [15]. This was followed by a proliferation of graph-based neural networks (GNNs), such as PiFold [16] and GeoSeqBuilder [17], which represent proteins as residue graphs to natively capture long-range spatial dependencies. Furthermore, the integration of AlphaFold 2 into design pipelines, exemplified by frameworks such as AlphaDesign, has enabled controllable, high-fidelity generation by closing the loop between design and structure validation [18].

Despite these advances, the quest for truly generalizable and robust design frameworks continues. Recent breakthroughs are pushing the boundaries of what is possible. For instance, the development of relaxed sequence optimization has challenged the traditional discrete nature of amino acids by formulating design as an optimization problem in a continuous sequence space, allowing for ultraefficient exploration and scalable generation of complex topologies [19]. Simultaneously, to combat data scarcity, particularly for functional protein generation, hierarchical models leveraging large language models, such as ProteinRG, have emerged [20]. These models utilize regenerative learning and pretrained sequence embeddings to generate high-quality, functional sequences even from small datasets. On the geometric front, novel architectures like ProtSeqGen are refining the precision of side-chain packing and local backbone compatibility through sophisticated multistage geometric graph networks, achieving superior sequence recovery rates across diverse structural subclasses [21].

Here, we introduce EquiDesign (Fig. 1), a framework for *de novo* protein sequence design employing Equiformer [22] as its core engine to model long-range spatial interactions. Input structures are encoded into local geometric graphs, extracting six complementary intra- and inter-residue matrices; Equiformer then performs spatially aware refinement by jointly optimizing invariant scalars and equivariant vectors. Trained on CATH 4.4 [23], EquiDesign outperformed state-of-the-art (SOTA) baselines on canonical (TS50/TS500) [16, 24] and challenging cohorts (IDRome-120 and TS45 [21]), exhibiting superior generalization. Notably, it generated thermodynamically stable, high-fidelity native-like folds across nine diverse proteins, establishing a robust platform for atomic-precision design of predefined scaffolds.

**Figure 1:**
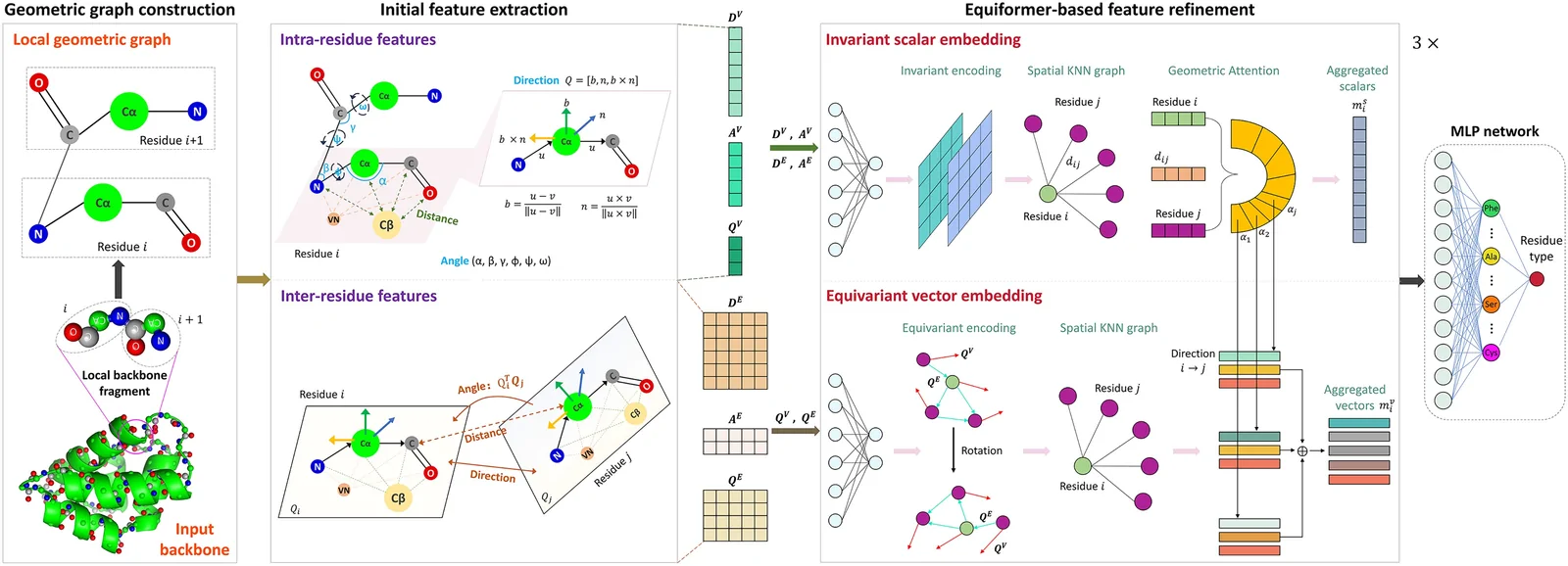
The overall architecture of EquiDesign. The initial from our previous work, ProtSeqGen [21].

## Materials and methods

### Dataset collection and preprocessing

Generalization across protein folds was assessed using the CATH 4.4 dataset [23], containing single-chain structures with a residue length between 40 and 500, enforcing a 40% sequence identity to eliminate homology redundancy. This yielded 29 453 training, 1732 validation, and 3466 test structures [25]. Model performance was further evaluated on the backbone-only benchmarks: TS50 and TS500, comprising 50 and 500 proteins with experimental structures [13, 26].

To probe challenging targets, we tested specialized datasets enriched for intrinsically disordered regions (IDRs) [27, 28] and free modeling (FM) targets [29, 30]. Curated from 1508 annotated candidates (<200 residues), IDRoom-120 [21] comprises 120 samples evenly distributed across four chain length intervals (<50, 51–100, 101–150, and 151–200), with exactly 30 samples in each category. Second, we employed TS45 (CASP15), a stringent benchmark of FM targets lacking detectable homology to known structures [29, 31]. Dataset statistics are summarized in Table 1.

**Table 1:** Description of datasets used in this study

Datasets	Total samples	Training	Validation	Unseen testing	Similarity
CATH 4.4	34 651	29 453	1732	3466	-
TS500	500	-	-	243	61.72%
TS50	50	-	-	26	75.83%
IDRome-120	120	-	-	120	61.86%
TS45	45	-	-	45	40.52%

Sequence similarity between each independent set and CATH 4.4 was calculated using MMseqs2.

### EquiDesign modeling

#### Generation of the local geometric graph

To effectively model local conformational details, we segment the input protein backbone into overlapping structural units (denoted as local backbone fragments in Fig. 1), each encompassing two adjacent residues [32]. For every such unit, a residue-level geometric graph is constructed to serve as the basis for downstream feature extraction, including intra- and inter-residue features [16, 17].

#### Initial feature extraction at the residue scale

This subsection describes three components inherited from ProtSeqGen [21]: local orthogonal basis definition, intra-residue feature generation, and inter-residue feature generation.

(1) Definition of local orthogonal basis

For each residue *i*, a local orthogonal basis ${Q}_i$ is established from the global coordinates of its three backbone atoms: nitrogen (${N}_{\boldsymbol{i}}$), carbon (${C_{\alpha}}_i$), and carbonyl carbon (${C}_{\boldsymbol{i}}$), with ${N}_{\boldsymbol{i}},{C_{\alpha}}_i,{C}_{\boldsymbol{i}}\in{R}^3$. We first construct three directional vectors directly from these coordinates


(1) \begin{equation*} b={C_{\alpha}}_i-{N}_{\boldsymbol{i}},c={C}_{\boldsymbol{i}}-{C_{\alpha}}_i,a=b\times c \end{equation*}

Their corresponding unit vectors are then obtained by normalization


(2) \begin{equation*} \overrightarrow{b}=\frac{b}{\parallel b\parallel },\overrightarrow{a}=\frac{a}{\parallel a\parallel },\overrightarrow{c}=\hat{b}\times \hat{a} \end{equation*}

The resulting basis ${Q}_i=\left[\overrightarrow{b},\overrightarrow{a},\overrightarrow{c}\right]$ is subsequently used to project atomic coordinates into the local frame, yielding the geometric features required for node- and edge-level computations.

(2) Generating the intraresidue features.

To precisely characterize local residue geometry, features are extracted from backbone atoms ($N,{C}_{\alpha },C,O$) and the $\mathrm{C}\mathrm{\beta}$ atom. Since the $\mathrm{C}\mathrm{\beta}$ atom plays a critical role in determining side-chain conformation, its coordinates computed via standard peptide geometry from the local *N*/${C}_{\alpha }$/*C* frame [33, 34], are included as key features. For glycine, a virtual $\mathrm{C}\mathrm{\beta}$ is generated identically to ensure consistency. The *Cβ* geometry is computed as:


(3) \begin{equation*} {C\beta}_i=-0.58273431\overrightarrow{a}+0.56802827\overrightarrow{b}-0.54067466\overrightarrow{c}+{C_{\alpha}}_i \end{equation*}

To capture intraresidue geometry, we introduce atom-level virtual nodes (vns) parameterized within the local frame. These nodes serve as abstract anchors to enhance graph representation, following prior work on attention mechanisms [35, 36].

The position of vn *g* is computed as:


(4) \begin{equation*} {\mathrm{vn}}_g={v}_x\bullet \overrightarrow{a}+{v}_y\bullet \overrightarrow{b}+{v}_z\bullet \overrightarrow{c}+\overrightarrow{r} \end{equation*}

where $\overrightarrow{r}$ is the ${C}_{\alpha }$ position vector. For residue *i*, we compute pairwise distances between its backbone atoms (${N}_i,{C_{\alpha}}_i,{C}_{\boldsymbol{i}},{O}_{\boldsymbol{i}}$), ${\mathrm{C}\mathrm{\beta}}_i$, and vn (${\mathrm{vn}}_{i,1}$,…${\mathrm{vn}}_{i,p},\dots{\mathrm{vn}}_{i,q},\dots$) within the local frame. These distances are encoded via a radial basis function (RBF) kernel to yield the distance feature vector ${D}_i^V$:


(5) \begin{align*} {D}_i^V=\left[\mathrm{RBF}\left(\left\Vert{N}_i-{C_{\alpha}}_i\right\Vert \right),\dots, \mathrm{RBF}\left(\left\Vert{C\alpha}_{\boldsymbol{i}}\ {O}_{\boldsymbol{i}}\right\Vert \right),\dots, \mathrm{RBF}\left(\left\Vert{\mathrm{vn}}_{i,p}-{\mathrm{vn}}_{i,q}\right\Vert \right)\right] \nonumber\end{align*}

The angle feature vector ${A}^V$ is derived from both dihedral and local angles. The three dihedral angle ($\varphi, \psi, \omega$) are defined by four consecutive atoms (*E,F,G,H*), calculated as


(6) \begin{equation*} f\left(E,F,G,H\right)=\arctan 2\left(\left({\overrightarrow{n}}_2\times{\overrightarrow{n}}_1\right)\cdotp{\overrightarrow{s}}_2,\kern0.5em {\overrightarrow{n}}_1\cdotp{\overrightarrow{n}}_2\Big)\cdotp \parallel{\overrightarrow{s}}_2\parallel \right) \end{equation*}

where ${\overrightarrow{s}}_1=F-E$, ${\overrightarrow{s}}_2=G-F$, ${\overrightarrow{s}}_3=H-G$ and ${n}_1={\overrightarrow{s}}_1\times{\overrightarrow{s}}_2$, ${n}_2={\overrightarrow{s}}_2\times{\overrightarrow{s}}_3$. Specifically, $\varphi =f\left({C}_{\boldsymbol{i}-\mathbf{1}},{N}_i,{C_{\alpha}}_i,{C}_{\boldsymbol{i}}\right)$, $\psi =f\left({N}_i,{C_{\alpha}}_i,{C}_{\boldsymbol{i}},{N}_{i+1}\right)$, and $\omega =f\ \left({C_{\alpha}}_i,{C}_{\boldsymbol{i}},{N}_{i+1},{C_{\alpha}}_{i+1}\right)$.

The three bond angles ($\alpha, \beta, \gamma$) are computed using the following expression:


(7) \begin{equation*} \left(E,F,G\right)=\operatorname{arccos}\left({\overrightarrow{u}}_0\cdotp{\overrightarrow{u}}_1\right) \end{equation*}

with ${\overrightarrow{u}}_0=\left(E-F\right)/\parallel E-F\parallel$ and ${\overrightarrow{u}}_1=\left(G-F\right)/\parallel G-F\parallel$. This yields $\alpha =l\left({N}_i,{C_{\alpha}}_i,{C}_{\boldsymbol{i}}\right)$, $\beta =l\left({C_{\alpha}}_i,{C}_{\boldsymbol{i}},{N}_{i+1}\right)$, and $\gamma =l\left({C}_{\boldsymbol{i}},{N}_{i+1},{C_{\alpha}}_{i+1}\right)$.

${A}_i^V$ is constructed as the sine and cosine encodings of all six angles:


(8) \begin{equation*} {A}_i^V=\left[\cos \left(\varphi \right),\sin \left(\varphi \right),\dots, \cos \left(\gamma \right),\sin \left(\gamma \right)\right] \end{equation*}

For each internal backbone atom $t\in \left(N,C,O\right)$ in residue *i*, we compute its vector relative to ${C_{\alpha}}_i$ and project it onto the local orthonormal basis $\left[\overrightarrow{a},\overrightarrow{b},\overrightarrow{c}\right]$ Equation (2). These projected coordinates are normalized to unit vectors, and their concatenation forms the direction feature ${Q}_i^V$:


(9) \begin{equation*} {Q}_i^V=\left[\frac{v_N}{\left\Vert{v}_N\right\Vert },\frac{v_C}{\left\Vert{v}_C\right\Vert },\frac{v_O}{\left\Vert{v}_O\right\Vert}\right] \end{equation*}

where ${\overrightarrow{v}}_t=\left(\left({t}_{\boldsymbol{i}}-{C_{\alpha}}_i\right)\bullet \overrightarrow{a},\kern0.5em \left({t}_{\boldsymbol{i}}-{C_{\alpha}}_i\right)\bullet \overrightarrow{b},\kern0.5em \left({t}_{\boldsymbol{i}}-{C_{\alpha}}_i\right)\bullet \overrightarrow{c}\right)$.

(3) Generating the inter-residue features

Inter-residue geometric features, namely distance ${D}^E$, angle ${A}^E$, and direction ${Q}^E$, are derived for each residue pair $\left(i,j\right)$. The distance feature ${D}_{ij}^E$ is obtained by applying an RBF kernel to the Euclidean distances between constituent atoms of residues *i* and *j*:


(10) \begin{equation*} {D}_{ij}^E=\left[\mathrm{RBF}\left(\left\Vert{m}_i-{m}_j\right\Vert \right),\dots, \mathrm{RBF}\left(\left\Vert{\mathrm{vn}}_i-{\mathrm{vn}}_j\right\Vert \right)\right] \end{equation*}

where ${m}_i\in \left({N}_i,{C_{\alpha}}_i,{C}_{\boldsymbol{i}},{O}_{\boldsymbol{i}},{C\beta}_i\right)$ and ${m}_j\in \left({N}_j,{C_{\alpha}}_j,{C}_{\boldsymbol{j}},{O}_{\boldsymbol{j}},{C\beta}_j\right)$, with ${\mathrm{vn}}_i$ and ${\mathrm{vn}}_j$ denoting vns from residues *i* and *j*, respectively.

The angle feature ${A}^E$ captures the relative 3D orientation between local frames via the rotation matrix ${R}_{ij}={Q}_i^T{Q}_j$, which aligns residue ${i}^{{\prime}}$s frame to residue ${j}^{{\prime}}$s frame. This matrix is converted to a unit quaternion ${A}^E=\left(w,x,y,z\right)$ using the trace-based method:


(11) \begin{align*} w&=0.5\sqrt{1+ Tr\ \left({R}_{ij}\right)},x=\frac{R_{ij}\left(3,2\right)-{R}_{ij}\left(2,3\right)}{4w},\nonumber\\y&=\frac{R_{ij}\left(1,3\right)-{R}_{ij}\left(3,1\right)}{4w},z=\frac{R_{ij}\left(2,1\right)-{R}_{ij}\left(1,2\right)}{4w} \end{align*}

The direction feature ${Q}_{ij}^E$ encodes the orientation of residue *j*’s backbone atoms relative to residue *i*’s ${C_{\alpha}}_i$. For each atom ${m}_j\in \left\{{N}_j,{C_{\alpha}}_i,{C}_j,{O}_j\right\}$, we compute its displacement vector from ${C_{\alpha}}_i$, project it onto residue ${i}^{{\prime}}$s local frame $\left[\overrightarrow{a},\overrightarrow{b},\overrightarrow{c}\right]$, and normalize:


(12) \begin{equation*} {Q}_{ij}^E=\left[\frac{e_N}{\left\Vert{e}_N\right\Vert },\frac{e_{C\alpha}}{\left\Vert{e}_{C\alpha}\right\Vert },\frac{e_C}{\left\Vert{e}_C\right\Vert },\frac{e_O}{\left\Vert{e}_O\right\Vert}\right] \end{equation*}

#### Feature refinement through the Equiformer network

The Equiformer network refines six geometric feature matrices, comprising intra-residue distances ${D}^V$, angles ${A}^V$, and directions ${Q}^V\in{R}^{101}$, alongside inter-residue distances ${D}^E$, angles ${A}^E$, and directions ${Q}^E\in{R}^{412}$.

(1) Invariant scalar embedding

Step 1: Invariant encoding

Raw geometric invariants, including inter-residue distances in ${D}^E$ and intraresidue bond angles in ${A}^V$, are linearly projected into high-dimensional node and edge representations, which serve as the latent features for subsequent geometric message passing:


(13) \begin{equation*} {h}_i^{{\prime}}=\mathrm{ScalarLinear}\left(\mathrm{Concate}\left({D}^V,{A}^V,{D}^E,{A}^E\right)\right) \end{equation*}

Step 2: Spatial K-nearest neighbor (KNN) graph

Using inter-residue distances (${d}_{ij}$), we construct a KNN graph to define the local neighborhood $\mathcal{N}(i)$ for each central residue *i*. This design mimics the biological tendency for interactions among spatially proximate residues (e.g. within local folding cores), thereby establishing the geometric context for message passing.


(14) \begin{equation*} \mathcal{N}(i)=\mathrm{Top}K\_\left(j\ne i\right)\ \left({d}_{ij}\;\right) \end{equation*}

Step 3: Geometric attention aggregation

Scalar messages propagate along edges, with attention weights modulated by edge features (distances ${d}_{ij}$, angles ${\theta}_{ij}$) and node features, enabling geometric awareness.

First, the attention input ${z}_{ij}$ is assembled by concatenating the central node scalar ${h}_i^{\prime }$, the neighboring node scalar ${h}_j^{\prime }$, the edge distance ${d}_{ij}$, and the edge angle ${\theta}_{ij}$:


(15) \begin{equation*} {z}_{ij}=\left[\;{h}_i^{\prime}\;\left\Vert{h}_j^{\prime}\;\right\Vert{d}_{ij}\;\Big\Vert{\theta}_{ij}\;\right] \end{equation*}

Second, unnormalized attention scores ${e}_{ij}$ are computed via an Multi-Layer Perceptron (MLP) applied to the concatenated vector ${z}_{ij}$:


(16) \begin{align*} {e}_{ij}=\mathrm{MLP}\left({z}_{ij}\right) \end{align*}

Third, attention weights ${\alpha}_{ij}$ are derived via softmax normalization over the local neighborhood $\mathcal{N}(i)$, with nonneighboring nodes masked to *-∞* to restrict aggregation to local contexts:


(17) \begin{equation*} {\alpha}_{ij}=\frac{\exp \left({e}_{ij}\right)}{\sum_{k\in \mathcal{N}(i)}\exp \left({e}_{ik}\right)} \end{equation*}

Finally, the refined scalar feature ${m}_i^s$ is aggregated via a weighted sum over neighbors using attention weights ${\alpha}_{ij}$, integrating local geometric context:


(18) \begin{equation*} {m}_i^s=\sum_{j\in \mathcal{N}(i)}{\alpha}_{ij}\bullet{h}_j^{\prime } \end{equation*}

(2) Equivariant vector embedding

Complementary to the scalar stream, this channel processes directional features (${Q}^V$, ${Q}^E$) to ensure that outputs rotate commensurately with the input structure. These features are first projected through an equivariant linear layer into high-dimensional vector representations, thereby preserving the geometric meaning of local orientations:


(19) \begin{equation*} {v}_i^{\prime }=\mathrm{VectorLinear}\left(\mathrm{Concat}\left({Q}^V,{Q}^E\right)\right) \end{equation*}

Message passing occurs over the same spatial KNN graph $\mathcal{N}(i)$ defined by inter-residue distances, ensuring geometric consistency between the invariant and equivariant pathways. Edge messages ${e}_{ij}$ are constructed by transforming neighbor vectors ${v}_j^{\prime }$ based on relative directions ${q}_{ij}$:


(20) \begin{equation*} {e}_{ji}=\mathrm{VectorTransform}\left({v}_j^{\prime },{q}_{ij}\right) \end{equation*}

Finally, the refined vector feature ${m}_i^v$ is aggregated via a weighted sum over neighbors using the attention weights ${\alpha}_{ij}$ derived from the scalar channel, maintaining strict rotation equivariance:


(21) \begin{equation*} {m}_i^v=\sum_{j\in \mathcal{N}(i)}{\alpha}_{ij}\bullet{e}_{ji} \end{equation*}

#### Residue type prediction with MLP classifier

The refined residue embedding ${h}_i^{(N)}$ is passed to an MLP classifier for amino acid type prediction (Fig. 1). The classifier outputs a 20-dimensional logit vector ${y}_i\in{\mathrm{R}}^{20}$, formulated as


(22) \begin{equation*} {y}_i=\mathrm{MLP}\left({h}_i^{(N)}\right) \end{equation*}

The predicted residue type ${\hat{Y}}_i$ is derived by applying the SoftMax function to ${y}_i$ and selecting the class with maximal probability


(23) \begin{equation*} {\hat{Y}}_i=\underset{l=\left\{1,\dots, 20\right\}}{\mathrm{argmax}}\left({\sigma}_l\left({y}_i\right)\right),\ \textrm{where}\ {\sigma}_l\left({y}_i\right)=\frac{e_{y_{i,l}}}{\sum_{j=1}^{20}{e}_{y_{i,j}}} \end{equation*}

Under this deterministic decoding, exactly one sequence is generated per input backbone, and this single sequence is directly reported as the final design without further selection.

### EquiDesign model training and calibration

Optimized using Adam with weight decay and label smoothing, EquiDesign employs a Noam scheduler with linear warm-up to ensure stable convergence [37]. Training utilizes automatic mixed precision and gradient clipping, with a fixed seed guaranteeing reproducibility. Manual hyperparameter tuning (Supplementary Tables S1 and S2) precedes evaluation, where final results are quantified as the mean and standard deviation across 10 independent training trials.

### Evaluation metrics

We evaluate EquiDesign using six metrics across sequence, structure, and modeling fidelity. Sequence recovery and perplexity quantify native sequence congruence [16]. Root-mean-square deviation (RMSD) and Structure similarity (TM-score) assess structural deviation from target backbones, providing complementary metrics for local precision and global fold accuracy [17]. Folding stability measures whether a generated sequence exhibits both high naturalness and proper folding propensity [21]. The loss function monitors training convergence. These metrics are defined as follows:


(24) \begin{equation*} \mathrm{Recovery}=\frac{1}{N}\sum_{i=1}^Nf\left({s}_i^d={s}_i^n\right) \end{equation*}

(25) \begin{equation*} \mathrm{Perplexity}=\exp \left(-\frac{1}{N}\sum_{i=1}^N\log P\left({s}_i|{s}_1,{s}_2,\dots, {s}_{i-1}\right)\right) \end{equation*}

(26) \begin{equation*} \mathrm{RMSD}=\sqrt{\frac{1}{N}\sum_{i=1}^N{\left\Vert{r}_i^d-{r}_i^n\right\Vert}^2} \end{equation*}

(27) \begin{equation*} \mathrm{TM}-\mathrm{score}=\max \left(\frac{1}{L_n}\sum_{i=1}^{L_a}\frac{1}{1+{\left({d}_i/\left({d}_0\left({L}_n\right)\right)\right)}^2}\right) \end{equation*}

(28) \begin{equation*} \mathrm{Stability}=0.86\bullet p\left(\mathrm{sequence}\right)+0.14\bullet p\left(\mathrm{structure}|\mathrm{sequence}\right) \end{equation*}

(29) \begin{equation*} \mathrm{Loss}=-\frac{1}{N}\sum_{i=1}^N\sum_{a=1}^{20}{y}_{i,a}\log \left({p}_{i,a}\right) \end{equation*}

Here, *N* denotes sequence length ($S=\left({s}_1,{s}_2,\dots, {s}_N\right)$); ${s}_i^d$ and ${s}_i^n$ are designed and native residues. $f\left(\ast \right)$ is a Boolean indicator; $P\left({s}_i|{s}_1,{s}_2,\dots, {s}_{i-1}\right)$ is the conditional probability. ${r}_i^d$ and ${r}_i^n$ are 3D atomic coordinates; ${L}_n$ and ${L}_a$ are native and aligned lengths; diis the aligned distance; ${d}_0\left({L}_n\right)=1.24\sqrt{L_n-1.5}-1.8$. ${y}_{i,a}$ and ${p}_{i,a}$ are ground-truth and predicted probabilities. $p\left(\mathrm{sequence}\right)$ and $p\left(\mathrm{structure}|\mathrm{sequence}\right)$ denote ESM-2 pseudolikelihood and half-masked ESMFold predicted local distance difference test (pLDDT), respectively.

### Comparisons with existing methods

For a holistic assessment, EquiDesign is benchmarked against 16 representative baselines, categorized into five architectural families. The first two categories include MLP-based techniques (SPIN [13], SPIN2 [38], and Wang’s model [39]) and Convolutional Neural Network (CNN)-based approaches (SPROF [40], ProDCoNN [41], DenseCPD [42], and ScFold [24]). Given that GNN approaches represent the current mainstream, we further divide them into two subgroups: message-passing GNNs (ProteinMPNN [15], PiFold [16], GeoSeqBuilder [17], ProtSeqGen [21]), global-aware GNNs (GraphTrans [43], GVP [44], GCA [16], AlphaDesign [18]). Notably, SurFold is categorized separately, as it uniquely integrates both structural and surface features [45]. Considering the broader coverage of structures, all models are trained on CATH 4.4 and evaluated on four independent test sets: TS50, TS500, IDRome-120, and TS45 (Table 1). Additionally, the evaluation of folding stability is implemented on both CATH 4.4 and CATH 4.2 [46] for unbiased benchmarking.

### Ablation study

We systematically evaluated the EquiDesign framework through three ablation perspectives. First, we optimized architectural components by analyzing the structural configuration of $\mathrm{C}\mathrm{\beta}$ geometry and vn (Table 2). Second, we generated three variants (${M}_1$−${M}_3$) *by* sequentially removed (i) inter-residue features, (ii) intraresidue features, and (iii) vector embeddings, comparing them against the full model (Table 3). Finally, we assessed the impact of varying Equiformer configurations (${M}_4$−${M}_7$) on overall performance.

**Table 2:** Ablation study on the impact of *Cβ* geometry and virtual-node configuration on model performance

Condition	Virtual node	Recovery (%) $\boldsymbol{\uparrow}$	Perplexity $\boldsymbol{\downarrow}$	Loss $\boldsymbol{\downarrow}$
With $\mathrm{C}\mathrm{\beta}$ geometry	0	52.51	4.12	1.43
	1	54.68	4.04	1.40
	2	56.59	3.95	1.37
	3	55.83	4.02	1.39
Without $\mathrm{C}\mathrm{\beta}$ geometry	0	50.75	4.19	1.46
	1	52.13	4.11	1.43
	2	53.76	4.07	1.42
	3	53.24	4.09	1.43

**Table 3:** Ablation study of EquiDesign on CATH 4.4 dataset.${M}_1-{M}_7$ refer to the variants of EquiDesign

Variant	Condition	Recovery (%) $\boldsymbol{\uparrow}$	Perplexity $\boldsymbol{\downarrow}$	Loss $\boldsymbol{\downarrow}$
${M}_1$	w/o inter-residue features	30.73	8.37	2.59
${M}_2$	w/o intra-residue features	42.64	6.12	1.98
${M}_3$	w/o vector embedding	53.76	4.35	1.47
${M}_4$	K_neighbors = 24	53.58	4.38	1.48
${M}_5$	1-layer EquiFormer block	49.21	4.92	1.59
${M}_6$	2-layer EquiFormer block	54.92	4.18	1.43
${M}_7$	4-layer EquiFormer block	55.97	3.99	1.38
Full model	3-layer EquiFormer block	56.59	3.95	1.37

### Probabilistic sampling for diverse sequence generation

To assess the model's ability to explore diverse yet physically plausible sequence space beyond deterministic prediction, we modified the final MLP layer to output a probability distribution over 20 amino acids via softmax, rather than a single argmax prediction. During inference, we applied top-*k* sampling (*k=5*) to generate four distinct sequences per backbone, yielding 36 designed sequences in total across the nine test cases. For reproducibility, we used a base seed of 260 816 with four consecutive seeds per protein, incremented by 100 between targets.

The generated sequences were evaluated using four complementary metrics: (i) sequence identity to the native sequence, measuring deviation from memorization, (ii) pairwise Hamming distance among sampled sequences, quantifying intrabackbone diversity, (iii) KL divergence of amino acid distributions relative to the native set, assessing physicochemical plausibility, and (iv) foldability rate (self-consistency root mean square deviation, (scRMSD) < 2.0 Å), validated by both ESMFold and AlphaFold 3 (AF3), ensuring structural validity.

### Simulation environment

EquiDesign was implemented in PyTorch 2.4.1 and Python 3.10.10, running on a system equipped with a 12th Gen Intel® Core™ i9-12900K processor and 128 GB RAM. All simulations leveraged CUDA 12.1.105 for acceleration on an NVIDIA GeForce RTX A4000 GPU (32 GB VRAM). Three-dimensional structures were predicted using AF3 via the AlphaFold Server (https://alphafoldserver.com).

## Results

### Ablation studies on model architecture

To validate the design of our model architecture, we systematically dissected the contributions of structural configurations, feature representation components, and Equiformer hyperparameters through comprehensive ablation studies.

First, we assessed structural choices by evaluating various combinations of $\mathrm{C}\mathrm{\beta}$ geometry and vn. As shown in Table 2, incorporating *Cβ* geometry was pivotal, with the model achieving peak performance specifically when utilizing two vn.

Second, we quantified the impact of removing specific feature embeddings (Table 3). The removal of inter-residue features (${M}_1$) caused the most severe degradation, underscoring their essential role in capturing long-range dependencies. In contrast, excluding intra-residue features (${M}_2$) or vector embeddings (${M}_3$) led to milder declines (recovery rates of 42.64% and 53.76%, respectively), suggesting that scalar invariant features alone provide a sufficiently robust backbone for sequence generation, while geometric details offer supplementary benefits.

Finally, we evaluated varying Equiformer depths (Table 3). Deeper architectures improved stability, but the gain from two to four layers was marginal (${M}_5$−${M}_7$). The full model with three-layer blocks achieved an optimal trade-off between capacity and efficiency, yielding the highest recovery (56.59%), and the lowest loss (1.37).

Collectively, these results validate the proposed architectural design.

### Evaluating EquiDesign against SOTA methods on CATH 4.4

On the CATH 4.4 test set, we compare EquiDesign against SOTA methods categorized by architectural features (Table 4). Under the standard backbone-only input condition, EquiDesign achieves a sequence recovery rate of 56.59%, with the lowest perplexity (3.95) and loss (1.37), outperforming all competitors across every metric. It surpasses conventional baselines (MLP, CNN) and message-passing GNNs (ProteinMPNN, PiFold, GeoSeqBuilder) by a substantial margin, including a 9.06% improvement over ProteinMPNN.

**Table 4:** Performance evaluation on CATH 4.4 dataset (mean *±* SD)

Feature embedding	Model	Recovery (%) $\boldsymbol{\uparrow}$	Perplexity $\boldsymbol{\downarrow}$	Loss $\boldsymbol{\downarrow}$
MLP	SPIN	29.82 *±* 0.38	9.03 *±* 0.16	2.28 *±* 0.07
	SPIN2	32.15 *±* 0.42	8.51 *±* 0.17	2.15 *±* 0.08
	Wang’s model	32.27 *±* 0.36	8.43 *±* 0.15	2.10 *±* 0.07
CNN	SPROF	35.47 *±* 0.29	7.16 *±* 0.15	1.91 *±* 0.07
	ProDCoNN	35.61 *±* 0.32	7.04 *±* 0.16	1.86 *±* 0.07
	DenseCPD	45.53 *±* 0.36	5.57 *±* 0.16	1.47 *±* 0.07
	ScFold	54.27 *±* 0.12	4.12 *±* 0.01	1.42 *±* 0.002
Message-passing GNNs	ProteinMPNN	47.53 *±* 0.27	4.46 *±* 0.08	1.49 *±* 0.03
	PiFold	53.52 *±* 0.20	4.41 *±* 0.06	1.43 *±* 0.01
	GeoSeqBuilder	53.74 *±* 0.23	4.39 *±* 0.07	1.43 *±* 0.02
	ProtSeqGen	55.21 *±* 0.16	4.02 *±* 0.06	1.39 *±* 0.01
Global-aware GNNs	GraphTrans	37.58 *±* 0.32	6.19 *±* 0.14	1.73 *±* 0.06
	GVP	39.21 *±* 0.33	5.86 *±* 0.13	1.62 *±* 0.04
	GCA	41.52 *±* 0.29	5.17 *±* 0.09	1.56 *±* 0.03
	AlphaDesign	43.08 *±* 0.26	4.96 *±* 0.07	1.53 *±* 0.02
	EquiDesign	56.59 *±* 0.11	3.95 *±* 0.05	1.37 *±* 0.01
Structure + Surface encoder	SurFold (Full model)	62.93 *±* 1.04	2.84 *±* 0.04	1.05 *±* 0.02
	SurFold (Backbone + Side)	57.90 *±* 0.28	3.18 *±* 0.04	1.16 *±* 0.01
	SurFold (Backbone only)	35.09 *±* 0.07	7.76 *±* 0.02	2.05 *±* 0.003

In contrast, SurFold is uniquely designed to integrate backbone, side-chain, and surface features for sequence prediction [45]. While SurFold (Full model) achieves the best overall performance on CATH 4.4, this comparison reveals a critical discrepancy in input granularity. When restricted to backbone-only input (the standard protocol for inverse folding evaluation), SurFold’s performance drops markedly.

Thus, EquiDesign not only surpasses traditional GNN methods but also demonstrates greater robustness than SurFold under the standard single-backbone input setting, validating its capacity for high-quality sequence design without reliance on auxiliary structural features.

### Generalization evaluation on canonical benchmarks: TS50 and TS500

To assess the generalization ability of EquiDesign, we evaluated the pretrained model on two independent test sets, TS50 and TS500. After removing all overlapping proteins with the training set, the evaluation comprised 26 and 243 previously unseen samples for TS50 and TS500, respectively. As summarized in Tables 5 and 6, EquiDesign achieved recovery rates of 59.47% on TS50 and 61.08% on TS500, outperforming most SOTA methods. Notably, it substantially exceeded strong baselines such as ProteinMPNN (56.23% on TS50, 59.56% on TS500) and maintained the top rank across both benchmarks.

**Table 5:** Performance evaluation on unseen TS50 samples using models trained on CATH 4.4 (mean *±* SD)

Feature Embedding	Model	Recovery (%) $\boldsymbol{\uparrow}$	Perplexity $\boldsymbol{\downarrow}$	Loss $\boldsymbol{\downarrow}$
MLP	SPIN	31.18 *±* 0.44	9.02 *±* 0.16	2.28 *±* 0.05
	SPIN2	34.51 *±* 0.41	8.36 *±* 0.15	2.12 *±* 0.05
	Wang’s model	34.27 *±* 0.42	8.54 *±* 0.15	2.06 *±* 0.05
CNN	SPROF	40.53 *±* 0.34	7.27 *±* 0.12	1.91 *±* 0.04
	ProDCoNN	42.18 *±* 0.31	7.04 *±* 0.10	1.84 *±* 0.04
	DenseCPD	52.04 *±* 0.28	5.72 *±* 0.08	1.49 *±* 0.03
	ScFold	56.66 *±* 0.59	3.76 *±* 0.06	1.32 *±* 0.02
Message-passing GNNs	ProteinMPNN	56.23 *±* 0.19	3.80 *±* 0.05	1.34 *±*0.02
	PiFold	59.02 *±* 0. 12	3.75 *±* 0.06	1.29 *±* 0.02
	GeoSeqBuilder	58.63 *±* 0.15	3.74 *±* 0.04	1.31 *±* 0.03
	ProtSeqGen	58.79 *±* 0.13	3.79 *±* 0.05	1.29 *±* 0.02
	SurFold (Backbone only)	34.82 *±* 0.33	7.70 *±* 0.03	2.04 *±* 0.01
Global-aware GNNs	GraphTrans	43.56 *±* 0.36	5.52 *±* 0.09	1.46 *±* 0.03
	GVP	45.53 *±* 0.31	5.19 *±* 0.08	1.38 *±* 0.03
	GCA	48.82 *±* 0.25	4.64 *±* 0.07	1.27 *±* 0.04
	AlphaDesign	49.86 *±* 0.23	5.07 *±* 0.07	1.42 *±* 0.03
	EquiDesign	59.47 *±* 0.10	3.69 *±* 0.04	1.28 *±* 0.02

**Table 6:** Performance evaluation on unseen TS500 samples using models trained on CATH 4.4 (mean *±* SD)

Feature embedding	Model	Recovery (%) $\boldsymbol{\uparrow}$	Perplexity $\boldsymbol{\downarrow}$	Loss $\boldsymbol{\downarrow}$
MLP	SPIN	31.52 *±* 0.39	8.87 *±* 0.12	2.22 *±* 0.04
	SPIN2	38.03 *±* 0.32	8.17 *±* 0.13	2.01 *±* 0.03
	Wang’s model	37.58 *±* 0.33	8.24 *±* 0.12	2.05 *±* 0.03
CNN	SPROF	41.56 *±* 0.31	7.02 *±* 0.09	1.84 *±* 0.03
	ProDCoNN	43.57 *±* 0.28	6.69 *±* 0.11	1.77 *±* 0.03
	DenseCPD	57.23 *±* 0.19	5.52 *±* 0.08	1.43 *±* 0.02
	ScFold	59.38 *±* 0.19	3.63 *±* 0.08	1.29 *±* 0.02
Message-passing GNNs	ProteinMPNN	59.56 *±* 0.13	3.40 *±* 0.06	1.27 *±* 0.01
	PiFold	60.58 *±* 0.11	3.34 *±* 0.05	1.28 *±* 0.01
	GeoSeqBuilder	60.67 *±* 0.12	3.36 *±* 0.06	1.26 *±* 0.01
	ProtSeqGen	60.82 *±* 0.12	3.37 *±* 0.04	1.27 *±* 0.01
	SurFold (Backbone only)	38.19 *±* 0.12	6.96 *±* 0.02	1.94 *±* 0.01
Global-aware GNNs	GraphTrans	46.08 *±* 0.26	4.98 *±* 0.12	1.36 *±* 0.02
	GVP	51.06 *±* 0.22	4.02 *±* 0.07	1.37 *±* 0.02
	GCA	49.58 *±* 0.23	4.64 *±* 0.08	1.28 *±* 0.01
	AlphaDesign	50.84 *±* 0.19	4.84 *±* 0.08	1.31 *±* 0.01
	EquiDesign	61.08 *±* 0.09	3.32 *±* 0.03	1.23 *±* 0.01

Furthermore, we performed a rigorous statistical comparison between EquiDesign and four representative methods (ProteinMPNN, PiFold, GeoSeqBuilder, and ProtSeqGen) (Supplementary Table S3), using paired tests across TS50 and TS500. The results confirm that EquiDesign significantly outperforms the other four methods across all metrics, while its advantage over ProtSeqGen in loss does not reach statistical significance (Supplementary Tables S4 and S5). This analysis underscores the robustness of EquiDesign’s performance despite the close competition.

### Perresidue accuracy and prediction confidence

We next assessed the residue-level prediction fidelity of EquiDesign on the TS500 dataset by benchmarking against native protein sequences. EquiDesign demonstrates a balanced amino acid propensity, effectively accommodating the full spectrum of hydrophobic, aromatic, polar, and charged residues. This chemical diversity is reflected in the secondary structure-dependent accuracy, which aligns with established physicochemical principles (Fig. 2A).

**Figure 2:**
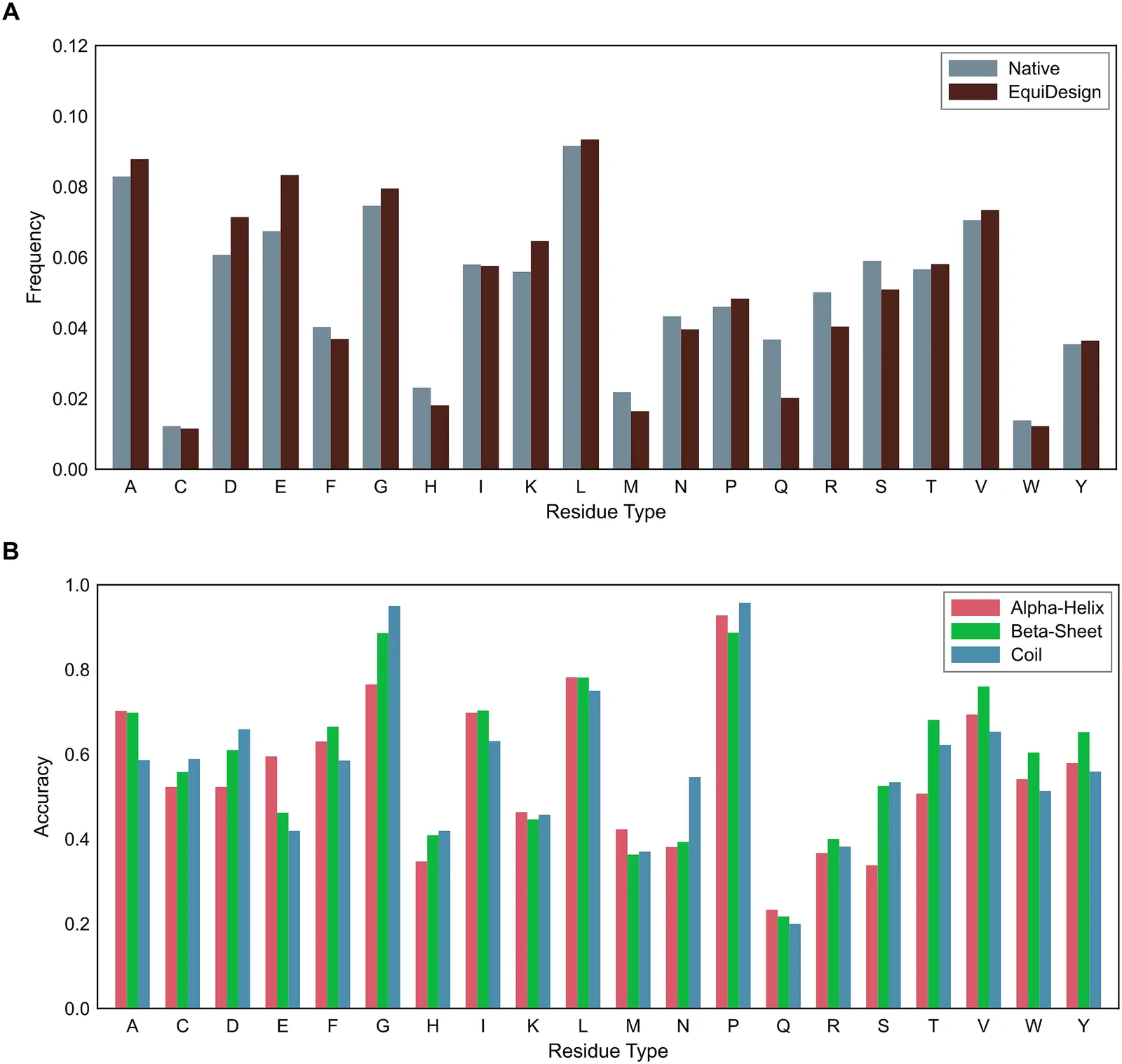
Impact of sequence redesign on *de novo* designed proteins (TS500). (A) Individual residue frequency between the native and predictions; (B) the prediction accuracy for central residue types for three secondary structures.

To quantify the contribution of local structural context, we parsed the recovery accuracy by residue type within alpha-helices, beta-sheets, and coils. As expected from their intrinsic structural biases, residues such as Thr (T), Val (V), Trp (W), and Tyr (Y) exhibit heightened recovery within beta-sheets, whereas Lys (K), Met (M), and Pro (P) are predicted with greater fidelity in alpha-helices and coils, respectively (Fig. 2B). Detailed per-residue accuracy metrics are provided in Supplementary Tables S6 and S7.

### Generalization evaluation on challenging benchmarks: IDRome-120 and TS45

To assess the capability of our model on challenging tasks, we applied the pretrained EquiDesign to specialized datasets featuring IDRs [27, 28] and FM targets [29].

First, we introduced the IDRome-120 dataset [21], comprising 120 proteins of diverse sequence lengths (<200 residues). As shown in Fig. 3A, EquiDesign delivered significantly superior performance in generating sequences for disordered regions. While the predictive performance of all evaluated methods declined relative to the standard TS50 and TS500 benchmarks, EquiDesign exhibited the smallest degradation, maintaining a substantial lead over competitors (e.g. ~3.6% higher recovery than ProtSeqGen).

**Figure 3:**
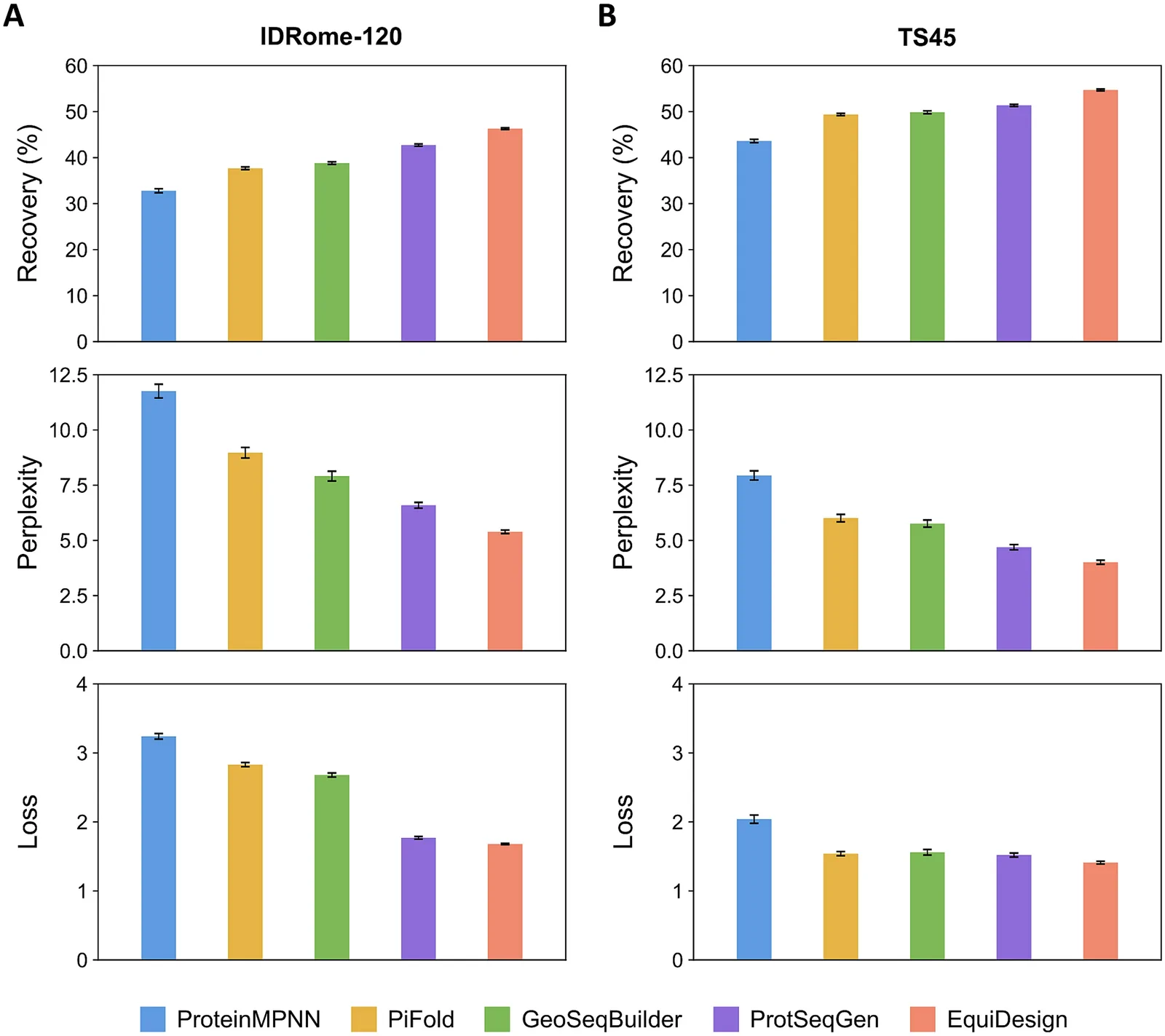
Performance comparison between EquiDesign and baseline models on the IDRome-120 and TS45 datasets, evaluated by recovery, perplexity, and loss.

Second, we evaluated the model on TS45, a highly challenging benchmark for *de novo* protein design consisting of FM targets with no homology to known structures [31]. The results on TS45 closely mirrored those on IDRome-120, with EquiDesign again achieving the top recovery rate (54.71%) (Fig. 3B). This consistency underscores the comparable difficulty of these two challenging benchmarks and highlights the robustness of EquiDesign under diverse and difficult design conditions (Supplementary Table S8).

### *De novo* protein design with EquiDesign

To rigorously evaluate the capability of EquiDesign in *de novo* sequence generation, we curated a benchmark suite of nine representative proteins spanning diverse structural classes (Table 7), including all-*α*, all-*β*, $\alpha /\beta$, $\alpha +\beta$ folds, and complex integral membrane proteins, covering a gradient of topological complexity from simple motifs to intricate TIM-barrels.

**Table 7:** Detailed information of nine representative proteins used for benchmarking

No.	PDB ID	Classification	Subclass
1	1f89.A	All-α	*Coiled coil*
2	1luc.A	All-β	Jelly roll
3	1n27.A	α + β	Zinc finger-like fold
4	1i07.A	α + β	Zinc finger-like fold
5	1a4i.A	α/β	TIM beta/alpha-barrel
6	1xe7.A	α/β	TIM beta/alpha-barrel
7	3u5t.A	α/β	TIM beta/alpha-barrel
8	2qk2.A	Membrane proteins	Voltage-gated ion channels-like superfamily
9	3h36.A	Membrane proteins	G protein-coupled receptor-like superfamily

EquiDesign generates sequences with high similarity to native sequences. Notably, seven out of the nine predicted sequences achieved a sequence recovery rate exceeding 55% (Table 8). To validate structural fidelity, we employed AF3 to predict the 3D structures of the generated sequences. As shown in Fig. 4, the predicted conformations exhibit remarkable structural similarity to native structures, with high TM-scores and low RMSD values (average RMSD of 0.903 Å), confirming that EquiDesign robustly produces sequences that accurately fold into target native-like architectures.

**Table 8:** Comparative analysis of recovery, structural fidelity, and stability scores for inverse-folding approaches on nine representative proteins

Protein	Method	$\mathbf{\uparrow}$ **Recovery (%)**	$\mathbf{\uparrow}$ **TM-score**	$\mathbf{\downarrow}$ **RMSD (**Å**)**	$\mathbf{\uparrow}$ **Stability score**
1f89.A	ProteinMPNN	47.26	0.694	1.64	9.233
	PiFold	51.82	0.825	2.54	7.131
	GeoSeqBuilder	50.72	0.858	1.54	8.340
	ProtSeqGen	51.65	0.852	1.52	7.893
	EquiDesign	53.17	0.860	1.49	9.563
1luc.A	ProteinMPNN	49.71	0.845	2.26	8.912
	PiFold	53.41	0.818	2.38	8.213
	GeoSeqBuilder	50.92	0.572	2.58	8.049
	ProtSeqGen	53.26	0.468	1.72	8.606
	EquiDesign	61.04	0.913	0.41	9.183
1n27.A	ProteinMPNN	46.85	0.254	5.31	9.234
	PiFold	54.39	0.645	3.14	8.856
	GeoSeqBuilder	53.74	0.656	2.43	8.324
	ProtSeqGen	55.21	0.885	1.52	9.306
	EquiDesign	56.27	0.889	1.46	9.611
1i07.A	ProteinMPNN	45.72	0.716	2.71	8.427
	PiFold	47.93	0.691	2.98	7.981
	GeoSeqBuilder	48.76	0.624	2.41	7.723
	ProtSeqGen	49.18	0.451	1.95	8.029
	EquiDesign	54.24	0.834	0.86	8.564
1a4i.A	ProteinMPNN	49.82	0.776	2.16	8.729
	PiFold	52.81	0.834	1.53	9.162
	GeoSeqBuilder	54.95	0.671	2.37	8.737
	ProtSeqGen	53.76	0.458	1.82	7.624
	EquiDesign	61.75	0.927	0.38	9.175
1xe7.A	ProteinMPNN	46.74	0.869	1.35	7.524
	PiFold	52.57	0.816	1.96	8.038
	GeoSeqBuilder	54.92	0.812	1.76	9.508
	ProtSeqGen	54.88	0.871	1.17	8.157
	EquiDesign	55.47	0.876	1.13	8.526
3u5t.A	ProteinMPNN	45.13	0.791	2.39	8.812
	PiFold	53.74	0.753	2.06	8.481
	GeoSeqBuilder	53.86	0.765	2.12	9.412
	ProtSeqGen	54.29	0.928	0.67	9.418
	EquiDesign	56.10	0.930	0.64	9.575
2qk2.A	ProteinMPNN	48.32	0.826	1.56	8.318
	PiFold	53.26	0.834	2.79	8.584
	GeoSeqBuilder	52.68	0.827	1.85	8.934
	ProtSeqGen	58.07	0.867	1.47	8.919
	EquiDesign	57.87	0.871	1.40	8.344
3h36.A	ProteinMPNN	43.67	0.563	2.80	8.129
	PiFold	50.83	0.644	3.51	8.148
	GeoSeqBuilder	51.11	0.559	2.81	8.881
	ProtSeqGen	55.13	0.898	0.38	8.468
	EquiDesign	56.47	0.904	0.36	8.370

**Figure 4:**
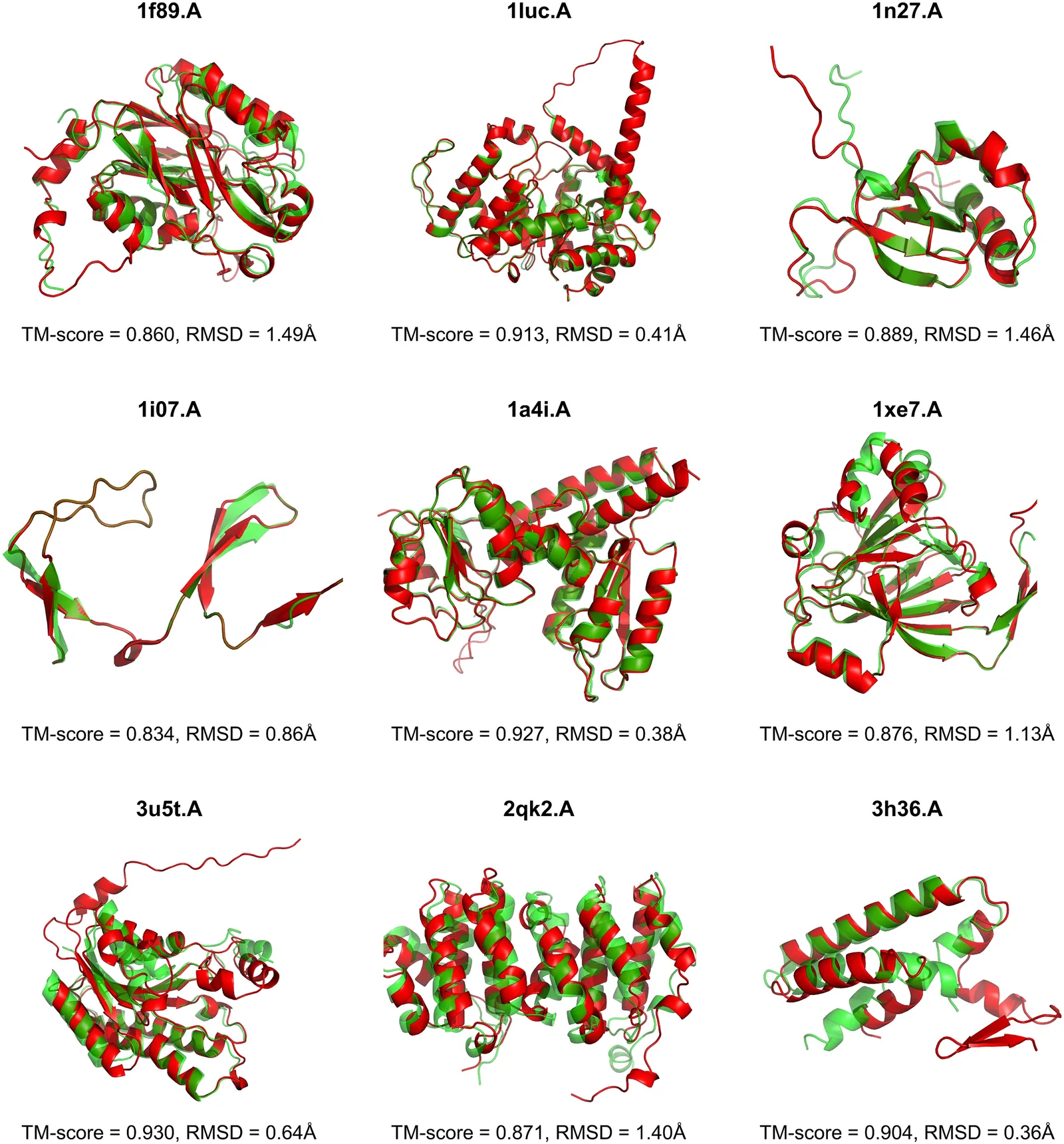
Overlay of native and designed protein structures. Green represents the native structures, while red shows the AlphaFold 3 predictions of the sequences generated by EquiDesign.

We further benchmarked EquiDesign against four SOTA models on this nonredundant dataset (Supplementary Figs S1–S4). As summarized in Table 8, EquiDesign consistently outperformed all comparators, achieving superior TM-scores and lower RMSD values. We also assessed the folding stability: designs for 1f89.A, 1luc.A, 1n27.A, 1i07.A, 1a4i.A, and 3u5t. A ranked first in stability among all evaluated models. In addition, statistical analyses showed that EquiDesign-generated sequences had significantly higher folding stability than two baseline methods, particularly ProteinMPNN and PiFold (Supplementary Table S9).

Moreover, Rosetta energy minimization confirmed that EquiDesign-designed sequences frequently achieve lower total energy than native sequences across the benchmark set, driven by reduced van der Waals repulsion (Fa_rep) and generally favorable attraction (Fa_atr), demonstrating thermodynamic stability beyond structural compatibility (Supplementary Table S10).

#### Diversity and novelty of generated sequences

To further assess whether EquiDesign explores viable alternative sequences beyond native recapitulation, we performed top-*k* sampling (*k=5*) to generate four sequences per backbone and evaluated them using four metrics (Supplementary Table S11). The sequence identity to native ranged from 35.2% to 65.2%, confirming the model’s ability to propose novel yet structurally compatible residues. Pairwise diversity (Hamming distance) averaged 15.3, indicating substantial sequence variation under the same backbone constraint. Critically, 50%–100% of the generated sequences were predicted to be foldable (scRMSD <2.0 Å) by both ESMFold and AF3. These results demonstrate that EquiDesign produces multiple distinct, physically plausible sequences for a given backbone, supporting its utility as a genuine design tool beyond residue classification.

Collectively, these results establish EquiDesign as a robust framework for generating sequences that faithfully fold into diverse native-like scaffolds, achieving exceptional precision and stability as validated by AF3.

## Discussion

In summary, we developed EquiDesign, evolving beyond our prior ProtSeqGen. The initial geometric feature extraction (including *Cβ* treatment), and vn components are adapted from ProtSeqGen. The key methodological novelty lies in replacing the conventional Message Passing Neural Network (MPNN) layers with an Equiformer-driven architecture for structure-guided sequence design. This upgrade enables explicit SE(3)-equivariant reasoning: whereas MPNNs implicitly propagate information via abstract node updates, EquiDesign disentangles features into invariant scalars and equivariant vectors, preserving directional backbone geometry during message passing. By leveraging spatial KNN graphs and geometric attention to fuse multiscale contextual cues, this framework achieves a more physically grounded representation of protein conformations, culminating in higher-fidelity sequence decoding.

Comprehensive ablation studies substantiate EquiDesign’s architectural efficacy. First, inter-residue features emerge as the most critical component, as their removal causes the most severe performance degradation, highlighting the importance of modeling long-range spatial dependencies is the fundamental challenge that conventional sequential GNNs fail to address. Second, integrating *Cβ* geometry with vn proves instrumental for further improvement, establishing a global information hub that transcends local sequential biases and elevates performance to the next level. Finally, hyperparameter optimization confirms that our configuration, specifically three-layer Equiformer blocks, achieves an optimal trade-off between model expressivity and computational efficiency. These findings validate EquiDesign as a physically principled advancement over prior graph-based paradigms.

EquiDesign converged stably on the CATH 4.4 benchmark (Supplementary Fig. S5), reflecting robust optimization with minimal overfitting. Notably, the pretrained model preserved high fidelity on held-out TS50 and TS500 sets; crucially, it simultaneously elevated predictive accuracy while reducing runtime and memory overhead relative to current SOTA counterparts (Supplementary Table S12). Moreover, EquiDesign exhibited remarkable generalization to high-difficulty regimes (IDRome-120 and TS45), particularly for IRDs and template-free targets, substantially surpassing representative baselines (Supplementary Tables S13 and S14). Collectively, these results position EquiDesign as a powerful framework capable of extending beyond well-folded proteins into structurally ambiguous landscapes.

Unlike conventional physics-based approaches, EquiDesign operates without task-specific parameter tuning, thereby democratizing access to high-fidelity protein design. Given diverse *de novo* backbones, the model generates sequences that consistently fold into target 3D structures with high accuracy, as evidenced by superior sequence recovery, TM-scores, and low RMSD values. Most critically, EquiDesign yields sequences with markedly enhanced stability, as validated by both redesign on nine diverse scaffolds (Table 8) and leading average stability scores across CATH 4.2/4.4 benchmarks (Supplementary Table S15). This capability to generate physically realistic, robust proteins significantly expands its practical utility.

To rigorously validate EquiDesign’s design principles beyond statistical recovery rates, we subjected the nine representative proteins to complementary structural and evolutionary analyses. First, we performed AlphaFold 3 (AF3) structure prediction on three sequence types (native, EquiDesign-predicted, and composition-matched scrambled sequences) and evaluated backbone fidelity via RMSD and confidence scores (pLDDT). EquiDesign-predicted sequences consistently recovered native-like structures with low RMSD (<2.0 Å) and high pLDDT values, whereas scrambled sequences universally failed to fold correctly, confirming that our pipeline generates physically plausible sequences rather than relying on amino acid composition alone (Supplementary Table S16). Second, to address potential memorization artifacts inherent to deep-learning-based structure predictors, we conducted homology searches (MMseqs2 [47]) and structural neighbor searches (Foldseek [48]) against the AF3 training set proxy (PDB entries released before September 30, 2021). While most targets exhibited high sequence identity (> 90%) with training structures, the maximum TM-scores for several proteins (e.g. 1i07.B at 0.926) were notably below 1.0, indicating that AF3 does not perfectly memorize specific backbone conformations (Supplementary Table S17). Critically, EquiDesign-generated sequences recapitulated target folds with high precision despite low sequence identity to training data. This confirms that our method captures genuine biophysical folding principles, not statistical correlations, and is robust against compositional biases and training set overfitting.

EquiDesign achieves superior inverse folding performance through its unified SE(3)-equivariant framework that synergistically integrates disentangled scalar–vector representations for simultaneous capture of invariant and directional features, and multiscale geometric attention for long-range topological reasoning, collectively enabling a more expressive and physically grounded latent representation than prior handcrafted or GVP-based approaches.

The current inverse folding paradigm has two key limitations: it depends on a prespecified backbone structure, and sequence recovery does not equate to functional activity. EquiDesign, like most structure-conditioned models, assumes the input backbone is optimal for the target function. However, this assumption may not hold in *de novo* design scenarios. Going forward, EquiDesign can be naturally integrated with generative backbone models (e.g. diffusion-based structure samplers) to form a complete design pipeline. Moreover, incorporating differentiable functional assays or experimental feedback loops could further close the gap between structural plausibility and biological activity.

Despite these strengths, EquiDesign presents several areas for further improvement. First, while effective for designing a diverse set of protein scaffolds (all-*α*, all-*β*, $\alpha /\beta$, $\alpha +\beta$ folds, membrane proteins, and TIM barrels), its transferability to other protein classes remains to be fully established. Second, the current study is limited by the lack of high-throughput structural validation of the generated protein designs. Lastly, training strategies tailored to distinct protein classes remain a crucial and open area for further investigation. To enhance the accessibility of EquiDesign, a user-friendly web server is currently under development and is planned for future release.

## Conclusion

We developed EquiDesign, an Equiformer-augmented framework that combines spatial KNN graphs with SE(3)-equivariant reasoning to integrate multiscale geometric features for structure-guided protein inverse folding. Benchmarking across CATH 4.4, TS50, TS500, IDRome-120, and TS45 demonstrates that EquiDesign achieves superior sequence recovery and robust generalization across diverse folds. While computational metrics are compelling, future work must prioritize rigorous experimental validation of the designed proteomes. Ultimately, EquiDesign establishes a powerful paradigm for *de novo* protein design, paving the way for accelerated development of next-generation therapeutics and biomaterials.

## Data and code availability

The data that support the findings of this study are publicly available from the following resources: the CATH database (version 4.4, http://www.cathdb.info), the standard benchmark datasets TS50/TS500, the IDRome-120 dataset [21], and the TS45 free-modeling targets [29]. The source code of EquiDesign is freely available on GitHub at https://github.com/NJAU-CDSIC/EquiDesign. In addition, the code for all SOTA baseline models and the complete benchmarking datasets (CATH 4.4, TS50, TS500, IDRome-120, and TS45) used in this study can be downloaded from the same repository.

**Key Points**

- Efficient and accurate *de novo* protein sequence design remains a challenge.
- It is appealing to develop novel computational approaches to improve the efficiency and accuracy​ of the *de novo* design of proteins.
- An Equiformer-augmented multiscale learning framework (EquiDesign) for protein sequence design was developed.
- EquiDesign outperformed 16 SOTA methods on benchmarking datasets.
- For various protein backbones, EquiDesign can design amino acid sequences that fold into the desired structure with high structural accuracy.

## Supplementary Material

Supplementary_Materials_bbag581

## References

[1] Gligorijevic V, Renfrew PD, Kosciolek T et al. Structure-based protein function prediction using graph convolutional networks. *Nat Commun* 2021;12:3168. 10.1038/s41467-021-23303-9 PMC8155034 34039967

[2] Xie S, Xie X, Zhao X et al. HNSPPI: a hybrid computational model combing network and sequence information for predicting protein-protein interaction. *Brief Bioinform* 2023;24:bbad261. 10.1093/bib/bbad261 37480553

[3] Song N, Li Z, Deng Y et al. MsipNet: a multi-scale representation learning framework for predicting protein-RNA interaction. *Int J Biol Macromol* 2026;339:149883. 10.1016/j.ijbiomac.2025.149883 41448304

[4] Mu J, Li Z, Zhang B et al. Graphormer supervised de novo protein design method and function validation. *Brief Bioinform* 2024;25:bbae135. 10.1093/bib/bbae135 PMC10982952 38557677

[5] Khoury GA, Smadbeck J, Kieslich CA et al. Protein folding and de novo protein design for biotechnological applications. *Trends Biotechnol* 2014;32:99–109. 10.1016/j.tibtech.2013.10.008 PMC3922204 24268901

[6] Xiong P, Chen Q, Liu H. Computational protein design under a given backbone structure with the ABACUS statistical energy function. *Methods Mol Biol* 2017;1529:217–26. 10.1007/978-1-4939-6637-0_10 27914053

[7] Norn C, Wicky BIM, Juergens D et al. Protein sequence design by conformational landscape optimization. *Proc Natl Acad Sci USA* 2021;118:e2017228118. 10.1073/pnas.2017228118 PMC7980421 33712545

[8] Listov D, Goverde CA, Correia BE et al. Opportunities and challenges in design and optimization of protein function. *Nat Rev Mol Cell Biol* 2024;25:639–53. 10.1038/s41580-024-00718-y PMC7616297 38565617

[9] Jacquin H, Gilson A, Shakhnovich E et al. Benchmarking inverse statistical approaches for protein structure and design with exactly solvable models. *PLoS Comput Biol* 2016;12:e1004889. 10.1371/journal.pcbi.1004889 PMC4866778 27177270

[10] Romero-Romero S, Lindner S, Ferruz N. Exploring the protein sequence space with global generative models. *Cold Spring Harb Perspect Biol* 2023;15:a041471. 10.1101/cshperspect.a041471 PMC10626256 37848247

[11] Koh H, Zheng Y, Yang M et al. AI-driven protein design. *Nat Rev Bioeng* 2025;3:1034–56. 10.1038/s44222-025-00349-8

[12] Peto M, Kloczkowski A, Honavar V et al. Use of machine learning algorithms to classify binary protein sequences as highly-designable or poorly-designable. *BMC Bioinformatics* 2008;9:487. 10.1186/1471-2105-9-487 PMC2655094 19014713

[13] Li Z, Yang Y, Faraggi E et al. Direct prediction of profiles of sequences compatible with a protein structure by neural networks with fragment-based local and energy-based nonlocal profiles. *Proteins* 2014;82:2565–73. 10.1002/prot.24620 PMC4177274 24898915

[14] Zhang J, Wang S, Gao L et al. Deep learning revolutionizes protein research: advances in structure prediction, functional annotation, and engineered design. *J Biotechnol* 2026;414:91–110. 10.1016/j.jbiotec.2026.03.012 41839238

[15] Dauparas J, Anishchenko I, Bennett N et al. Robust deep learning-based protein sequence design using ProteinMPNN. *Science* 2022;378:49–56. 10.1126/science.add2187 PMC9997061 36108050

[16] Gao Z, Tan C, Li S. Pifold: Toward effective and efficient protein inverse folding. In: Kiela D, Cho K, Oh A, (eds). International Conference on Learning Representations (ICLR 2023), Kigali, Rwanda: OpenReview.net, 2023. p. 1-14.

[17] Liu J, Guo Z, You H et al. All-atom protein sequence design based on geometric deep learning. *Angew Chem Int Ed Engl* 2024;63:e202411461. 10.1002/anie.202411461 39295564

[18] Jendrusch MA, Yang ALJ, Cacace E et al. AlphaDesign: a de novo protein design framework based on AlphaFold. *Mol Syst Biol* 2025;21:1166–89. 10.1038/s44320-025-00119-z PMC12405559 40527958

[19] Frank C, Khoshouei A, Fubeta L et al. Scalable protein design using optimization in a relaxed sequence space. *Science* 2024;386:439–45. 10.1126/science.adq1741 PMC11734486 39446959

[20] Ren C, He D, Huang J. De novo functional protein sequence generation: overcoming data scarcity through regeneration and large language models. *Brief Bioinform* 2026;27:bbag095. 10.1093/bib/bbag095 PMC12967336 41795657

[21] Gao Q, Li Z, Deng Y et al. ProtSeqGen: a novel deep learning model for protein sequence design. *BMC Bioinformatics* 2026;27:156. 10.1186/s12859-026-06482-4 PMC13390367 42192491

[22] Liao Y, Smidt T. Equiformer: equivariant graph attention transformer for 3D atomistic graphs. *ICLR* 2023;2023:1–32.

[23] Waman VP, Bordin N, Lau A et al. CATH v4.4: major expansion of CATH by experimental and predicted structural data. *Nucleic Acids Res* 2025;53:D348–55. 10.1093/nar/gkae1087 PMC11701635 39565206

[24] Zhong J, Zou Z, Qiu J et al. ScFold: a GNN-based model for efficient inverse folding of short-chain proteins via spatial reduction. *Brief Bioinform* 2025;26:bbaf156. 10.1093/bib/bbaf156 PMC11982017 40205854

[25] Yu G, Bi X, Ma T et al. CATH-ddG: towards robust mutation effect prediction on protein-protein interactions out of CATH homologous superfamily. *Bioinformatics* 2025;41:i362–72. 10.1093/bioinformatics/btaf228 PMC12261453 40662779

[26] Ingraham J, Garg V, Barzilay R et al. Generative models for graph-based protein design. In: Wallach H, Larochelle H, Beygelzimer A et al. (eds). Advances in *Neural Information Processing Systems 32 (NeurIPS 2019)*. Vancouver, Canada: Curran Associates, Inc.; 2019. p. 15820-31.

[27] Tesei G, Trolle AI, Jonsson N et al. Conformational ensembles of the human intrinsically disordered proteome. *Nature* 2024;626:897–904. 10.1038/s41586-023-07004-5 38297118

[28] Holehouse AS, Kragelund BB. The molecular basis for cellular function of intrinsically disordered protein regions. *Nat Rev Mol Cell Biol* 2024;25:187–211. 10.1038/s41580-023-00673-0 PMC11459374 37957331

[29] Kryshtafovych A, Rigden DJ. To split or not to split: CASP15 targets and their processing into tertiary structure evaluation units. *Proteins* 2023;91:1558–70. 10.1002/prot.26533 PMC10687315 37254889

[30] Simpkin AJ, Mesdaghi S, Sanchez Rodriguez F et al. Tertiary structure assessment at CASP15. *Proteins* 2023;91:1616–35. 10.1002/prot.26593 PMC10792517 37746927

[31] Liu M, Cheng X, Gao Z et al. ProtInvTree: deliberate protein inverse folding with reward-guided tree search. *NeurlPS* 2026;2026:1–15.

[32] Pereira J, Lamzin VS. A distance geometry-based description and validation of protein main-chain conformation. *IUCrJ* 2017;4:657–70. 10.1107/S2052252517008466 PMC5619857 28989721

[33] Jumper J, Evans R, Pritzel A et al. Highly accurate protein structure prediction with AlphaFold. *Nature* 2021;596:583–9. 10.1038/s41586-021-03819-2. PMC8371605 34265844

[34] Yang J, Anishchenko I, Park H et al. Improved protein structure prediction using predicted interresidue orientations. *Proc Natl Acad Sci USA* 2020;117:1496–503. 10.1073/pnas.1914677117 PMC6983395 31896580

[35] Cai C, Hy T, Yu R et al. On the connection between MPNN and graph transformer. In: Krause A, Brunskill E, Cho K et al. (eds). Proceedings of the 40th International conference on machine learning (ICML2023). Honolulu, Hawaii, USA: PMLR; 2023. p. 3408–30.

[36] Hwang E, Thost V, Sankar S et al. An analysis of virtual nodes in graph neural networks for link prediction. In: The first learning on graphs conference (IoG 2022); 9-12 Dec 2022; Virtual. OpenReview.

[37] Kalra D, Brakeshli M. Why warmup the learning rate? Underlying mechanisms and improvements. In: Globerson A, Mackey L, Belgrave D et al. (eds). Advances in Neural Information Processing Systems 37 (NeurIPS 2024). Vancouver, Canada: Curran Associates, Inc.; 2024. p. 111760-801.

[38] O'Connell J, Li Z, Hanson J et al. SPIN2: predicting sequence profiles from protein structures using deep neural networks. *Proteins* 2018;86:629–33. 10.1002/prot.25489 29508448

[39] Wang J, Cao H, Zhang JZH et al. Computational protein design with deep learning neural networks. *Sci Rep* 2018;8:6349. 10.1038/s41598-018-24760-x PMC5910428 29679026

[40] Chen S, Sun Z, Lin L et al. To improve protein sequence profile prediction through image captioning on pairwise residue distance map. *J Chem Inf Model* 2020;60:391–9. 10.1021/acs.jcim.9b00438 31800243

[41] Zhang Y, Chen Y, Wang C et al. ProDCoNN: protein design using a convolutional neural network. *Proteins* 2020;88:819–29. 10.1002/prot.25868 PMC8204568 31867753

[42] Qi Y, Zhang JZH. DenseCPD: improving the accuracy of neural-network-based computational protein sequence design with DenseNet. *J Chem Inf Model* 2020;60:1245–52. 10.1021/acs.jcim.0c00043 32126171

[43] Zhang X, Yin H, Ling F et al. SPIN-CGNN: improved fixed backbone protein design with contact map-based graph construction and contact graph neural network. *PLoS Comput Biol* 2023;19:e1011330. 10.1371/journal.pcbi.1011330 PMC10729952 38060617

[44] Jing B, Eismann S, Suriana P et al. Learning from protein structure with geometric vector perceptrons. In: International Conference on Learning Representations (ICLR 2021). Virtual: OpenReview; 2021.

[45] Li D, Shen L, Song M et al. SurfFold: a unified model for protein inverse folding by integrating surface and structural information. *Bioinformatics* 2026;42:btaf666. 10.1093/bioinformatics/btaf666 PMC12857571 41416920

[46] Sillitoe I, Dawson N, Lewis TE et al. CATH: expanding the horizons of structure-based functional annotations for genome sequences. *Nucleic Acids Res* 2019;47:D280–4. 10.1093/nar/gky1097 PMC6323983 30398663

[47] Steinegger M, Soding J. MMseqs2 enables sensitive protein sequence searching for the analysis of massive data sets. *Nat Biotechnol* 2017;35:1026–8. 10.1038/nbt.3988 29035372

[48] van Kempen M, Kim SS, Tumescheit C et al. Fast and accurate protein structure search with Foldseek. *Nat Biotechnol* 2024;42:243–6. 10.1038/s41587-023-01773-0 PMC10869269 37156916

