# Supplementary material for: EquiDesign: a versatile computational framework for de novo protein sequence design
Source: Brief Bioinform. 2026 Oct 26;27(5):bbag581. doi: 10.1093/bib/bbag581 (PMC13704781; doi:10.1093/bib/bbag581)
Supplement: Supplementary file 1 [file supplementary_materials_bbag581.docx]

**Supplementary Materials**

**EquiDesign: A Versatile Computational Framework for *De Novo***

**Protein Sequence Design**

Qiang Gao^1,2, #^, Zhongyu Xu^1,2, #^, Kai Chen^3^, Xiaochen Cen^1,2^, Ke Yan^4,5^, Jinxin Liu^6^, Zhiwei Ji^1,2,^ *

^1^College of Artificial Intelligence, Nanjing Agricultural University, No. 666 Binjiang Avenue, Nanjing, Jiangsu 211800, China

^2^Center for Data Science and Intelligent Computing, Nanjing Agricultural University, No. 666 Binjiang Avenue, Nanjing, Jiangsu 211800, China

^3^Research and development-Statistics, Eli Lilly and Company, Lilly Corporate Center, Indianapolis, IN 46285, USA

^4^Department of the Built Environment, College of Design and Engineering, National University of Singapore, 4 Architecture Drive, Singapore 117566, Singapore

^5^College of Mechanical and Vehicle Engineering, Hunan University, Changsha 410082, China

^6^Laboratory of Gastrointestinal Microbiology, College of Animal Science and Technology, Nanjing Agricultural University, No. 1 Weigang Rd., Nanjing 210095, China

^#^The authors contribute equally

*Corresponding author: Zhiwei Ji ([Zhiwei.Ji@njau.edu.cn](mailto:Zhiwei.Ji@njau.edu.cn))

**Supplementary Figures**

**Supplementary Fig. 1.** *De novo* protein designs by ProteinMPNN and their structural predictions. Native and predicted structures are shown in green and red, respectively.

**Supplementary Fig. 2.** *De novo* protein designs by PiFold and their structural predictions. Native and predicted structures are shown in green and red, respectively.

**Supplementary Fig. 3.** *De novo* protein designs by GeoSeqBuilder and their structural predictions. Native and predicted structures are shown in green and red, respectively.

**Supplementary Fig. 4.** *De novo* protein designs by ProtSeqGen and their structural predictions. Native and predicted structures are shown in green and red, respectively.

**Supplementary Fig. 5.** Convergence and performance assessment of EquiDesign on CATH 4.4. **(A)** the training loss curve; **(B)** the sequence recovery on validation set.

**Supplementary Tables**

**Supplementary Table 1.** The Hyperparameters of EquiDesign

| **Parameters** | **Value** |
| --- | --- |
| Max_protein_length | 10000 |
| Node_Features | 128 |
| Edge_Features | 128 |
| Optimal_virtual_atoms | 2 |
| Hidden_dim | 128 |
| K_neighbors | 48 |
| Num_EquiFormer_layers | 3 |
| Dropout | 0.32 |
| Optimizer | NoamOpt |
| Batch size | 10000 |
| Maximal Epoch | 700 |
| Augment_eps | 0.05 |
| Gradient_clip | 1.0 |

**Supplementary Table 2.** Internal parameters of EquiFormer Block

| **Parameters** | **Value** |
| --- | --- |
| In_scalar_dim | 128 |
| Out_scalar_dim | 128 |
| In_vector_dim | 16 |
| Out_vector_dim | 16 |
| Hidden_att | 128 |
| Edge_feat_dim | 128 |
| Edge_att_dim | 64 |
| Att_in_dim | 320 |
| Scalar_FFN_hidden | 512 |
| Dropout | 0.32 |

**Supplementary Table 3**. Default setting of key parameters of four representative SOTA models

| **Methods** | **Key parameters and defalt values** |
| --- | --- |
| ProtSeqGen | Max_protein_length = 10000 |
|  | Node_features = 128, Edge_features = 128 |
|  | Optimal_virtual_atoms = 2 |
|  | MPNN_hidden_dim = 128, MPNN_k_neighbors = 48, MPNN_attention_layer = 1 |
| GeoSeqBuilder | Num_scale_layers = 5, Hidden_units = 50 |
|  | Radius of contextual environment = 12Å |
|  | Node_features = 26, Edige_features = 345 |
| PiFold | Max_Epoch = 100, Learning_rate = 0.001, Batch_size = 8 |
|  | Hidden_dim = 128, PiGNN_layer = 10 |
|  | Encoder_layer = 10, Autoregressive_ layers = 0 |
| ProteinMPNN | Sampling temperature = 0.1, Backbone_noise = 0 |
|  | Encoder_layer = 3, Encoder_hidden_dim = 128 |
|  | Decoder_layer = 3 (vanilla MPNN), Decoder_hidden_dim = 128 |
|  | MLP_layer = 3 (GELU function) |

**Supplementary Table 4.** Statistical comparison on TS50 between EquiDesign and other methods (mean $\pm$ 95%CI)

| **Comparison** | **Metrics** | **Mean difference** | **95% CI** | **p-value** |
| --- | --- | --- | --- | --- |
| EquiDesign vs. ProteinMPNN | Recovery (%) $\boldsymbol{\uparrow}$ | +3.2412 | (3.1264, 3.3536) | 2.5998E-13 |
|  | Perplexity $\boldsymbol{\downarrow}$ | -0.1175 | (-0.1515, -0.0685) | 2.0314E-04 |
|  | Loss $\boldsymbol{\downarrow}$ | -0.0634 | (-0.0739, -0.0461) | 4.3752E-06 |
| EquiDesign vs. PiFold | Recovery (%) $\boldsymbol{\uparrow}$ | +0.4518 | (0.4074, 0.4926) | 1.8722E-09 |
|  | Perplexity $\boldsymbol{\downarrow}$ | -0.0639 | (-0.0965, -0.0235) | 4.8179E-03 |
|  | Loss $\boldsymbol{\downarrow}$ | -0.0128 | (-0.0208, 0.0008) | 6.5362E-02 |
| EquiDesign vs. GeoSeqBuilder | Recovery (%) $\boldsymbol{\uparrow}$ | +0.8463 | (0.7569, 0.9231) | 2.7863E-09 |
|  | Perplexity $\boldsymbol{\downarrow}$ | -0.0584 | (-0.0809, -0.0191) | 5.2044E-03 |
|  | Loss $\boldsymbol{\downarrow}$ | -0.0392 | (-0.0462, -0.0138) | 2.3204E-03 |
| EquiDesign vs. ProtSeqGen | Recovery (%) $\boldsymbol{\uparrow}$ | +0.6836 | (0.6263, 0.7337) | 3.7331E-10 |
|  | Perplexity $\boldsymbol{\downarrow}$ | -0.1283 | (-0.1262, -0.0718) | 1.7950E-05 |
|  | Loss $\boldsymbol{\downarrow}$ | -0.0126 | (-0.0233, 0.0037) | 1.2465E-01 |

**Supplementary Table 5.** Statistical comparison on TS500 between EquiDesign and other methods (mean $\pm$ 95%CI)

| **Comparison** | **Metrics** | **Mean difference** | **95% CI** | **p-value** |
| --- | --- | --- | --- | --- |
| EquiDesign vs. ProteinMPNN | Recovery (%) $\boldsymbol{\uparrow}$ | 1.5213 | (1.4328, 1.6072) | 2.1507E-11 |
|  | Perplexity $\boldsymbol{\downarrow}$ | -0.0697 | (-0.1062, -0.0338) | 1.7762E-03 |
|  | Loss $\boldsymbol{\downarrow}$ | -0.0421 | (-0.0448, -0.0352) | 1.5141E-08 |
| EquiDesign vs. PiFold | Recovery (%) $\boldsymbol{\uparrow}$ | 0.4879 | (0.4296, 0.5704) | 6.1789E-08 |
|  | Perplexity $\boldsymbol{\downarrow}$ | -0.0104 | (-0.0483, 0.0283) | 5.6912E-01 |
|  | Loss $\boldsymbol{\downarrow}$ | -0.0516 | (-0.0551, -0.0449) | 3.4827E-09 |
| EquiDesign vs. GeoSeqBuilder | Recovery (%) $\boldsymbol{\uparrow}$ | 0.4143 | (0.3344, 0.4856) | 6.3983E-07 |
|  | Perplexity $\boldsymbol{\downarrow}$ | -0.0296 | (-0.0683, 0.0078) | 1.0778E-01 |
|  | Loss $\boldsymbol{\downarrow}$ | -0.0308 | (-0.0347, -0.0254) | 2.8465E-07 |
| EquiDesign vs. ProtSeqGen | Recovery (%) $\boldsymbol{\uparrow}$ | 0.2673 | (0.1766, 0.3434) | 5.9824E-05 |
|  | Perplexity $\boldsymbol{\downarrow}$ | -0.0411 | (-0.0621, -0.0179) | 2.6901E-03 |
|  | Loss $\boldsymbol{\downarrow}$ | -0.0383 | (-0.0449, -0.0351) | 1.8961E-08 |

**Supplementary Table 6**. Amino acid composition of native versus EquiDesign-predicted sequences

| **Residue type** | **Native** | **Designed** | **Ratio in Native** | **Ratio in Designed** |
| --- | --- | --- | --- | --- |
| A | 10856 | 11492 | 0.0829 | 0.0878 |
| C | 1598 | 1507 | 0.0122 | 0.0115 |
| D | 7949 | 9347 | 0.0607 | 0.0714 |
| E | 8831 | 10903 | 0.0674 | 0.0833 |
| F | 5278 | 4830 | 0.0403 | 0.0369 |
| G | 9764 | 10412 | 0.0746 | 0.0795 |
| H | 3031 | 2371 | 0.0231 | 0.0181 |
| I | 7601 | 7542 | 0.058 | 0.0576 |
| K | 7315 | 8465 | 0.0559 | 0.0646 |
| L | 11990 | 12228 | 0.0916 | 0.0934 |
| M | 2860 | 2150 | 0.0218 | 0.0164 |
| N | 5676 | 5189 | 0.0433 | 0.0396 |
| P | 6025 | 6323 | 0.046 | 0.0483 |
| Q | 4812 | 2647 | 0.0367 | 0.0202 |
| R | 6561 | 5295 | 0.0501 | 0.0404 |
| S | 7732 | 6666 | 0.059 | 0.0509 |
| T | 7408 | 7607 | 0.0566 | 0.0581 |
| V | 9227 | 9618 | 0.0705 | 0.0734 |
| W | 1807 | 1601 | 0.0138 | 0.0122 |
| Y | 4639 | 4767 | 0.0354 | 0.0364 |

**Supplementary Table 7**. Prediction accuracy for residue types across three secondary structures (H, E, and C)

| **Residue**  **type** | **Alpha-Helix (H)** | | | **Beta-Sheet (E)** | | | **Coil (C)** | | |
| --- | --- | --- | --- | --- | --- | --- | --- | --- | --- |
|  | Native | Designed | Accuracy | Native | Designed | Accuracy | Native | Designed | Accuracy |
| A | 5468 | 3840 | 0.702 | 2054 | 1434 | 0.698 | 3334 | 1955 | 0.586 |
| C | 451 | 236 | 0.523 | 554 | 309 | 0.558 | 593 | 349 | 0.589 |
| D | 2529 | 1323 | 0.523 | 1129 | 689 | 0.61 | 4291 | 2828 | 0.659 |
| E | 4394 | 2614 | 0.595 | 1568 | 724 | 0.462 | 2869 | 1201 | 0.419 |
| F | 1795 | 1130 | 0.63 | 1888 | 1255 | 0.665 | 1595 | 933 | 0.585 |
| G | 1678 | 1284 | 0.765 | 1577 | 1398 | 0.886 | 6509 | 6181 | 0.95 |
| H | 948 | 329 | 0.347 | 751 | 307 | 0.409 | 1332 | 558 | 0.419 |
| I | 2696 | 1881 | 0.698 | 3157 | 2218 | 0.703 | 1748 | 1103 | 0.631 |
| K | 3026 | 1400 | 0.463 | 1476 | 658 | 0.446 | 2813 | 1286 | 0.457 |
| L | 5435 | 4252 | 0.782 | 3387 | 2644 | 0.781 | 3168 | 2376 | 0.75 |
| M | 1183 | 501 | 0.423 | 749 | 272 | 0.363 | 928 | 343 | 0.37 |
| N | 1544 | 588 | 0.381 | 938 | 369 | 0.393 | 3194 | 1745 | 0.546 |
| P | 1159 | 1076 | 0.928 | 680 | 603 | 0.887 | 4186 | 4005 | 0.957 |
| Q | 2203 | 514 | 0.233 | 933 | 202 | 0.217 | 1676 | 336 | 0.2 |
| R | 2798 | 1027 | 0.367 | 1536 | 614 | 0.4 | 2227 | 851 | 0.382 |
| S | 2315 | 783 | 0.338 | 1658 | 870 | 0.525 | 3759 | 2008 | 0.534 |
| T | 1943 | 985 | 0.507 | 2199 | 1498 | 0.681 | 3266 | 2032 | 0.622 |
| V | 2752 | 1909 | 0.694 | 4227 | 3213 | 0.76 | 2248 | 1469 | 0.653 |
| W | 717 | 388 | 0.541 | 533 | 322 | 0.604 | 557 | 286 | 0.513 |
| Y | 1548 | 897 | 0.579 | 1639 | 1069 | 0.652 | 1452 | 811 | 0.559 |

**Supplementary Table 8**. Performance evaluation on IDRome-120 and TS45 using models trained on CATH 4.4 (mean $\pm$ SD)

| **Dataset** | **Method** | **Recovery (%)** $\boldsymbol{\uparrow}$ | **Perplexity** $\boldsymbol{\downarrow}$ | **Loss** $\boldsymbol{\downarrow}$ |
| --- | --- | --- | --- | --- |
| IDRome-120 | ProteinMPNN | 32.79 ± 0.43 | 11.76 ± 0.31 | 3.24 ± 0.04 |
|  | PiFold | 37.68 ± 0.29 | 8.97 ± 0.24 | 2.83 ± 0.03 |
|  | GeoSeqBuilder | 38.82 ± 0.28 | 7.91 ± 0.22 | 2.68 ± 0.03 |
|  | ProtSeqGen | 42.72 ± 0.25 | 6.59 ± 0.13 | 1.77 ± 0.02 |
|  | EquiDesign | 46.30 ± 0.18 | 5.39 ± 0.08 | 1.68 ± 0.01 |
| TS45 | ProteinMPNN | 43.62 ± 0.37 | 7.94 ± 0.21 | 2.04 ± 0.06 |
|  | PiFold | 49.37 ± 0.25 | 6.01 ± 0.17 | 1.54 ± 0.03 |
|  | GeoSeqBuilder | 49.85 ± 0.32 | 5.76 ± 0.16 | 1.56 ± 0.04 |
|  | ProtSeqGen | 51.36 ± 0.23 | 4.69 ± 0.12 | 1.52 ± 0.03 |
|  | EquiDesign | 54.71 ± 0.19 | 4.01 ± 0.09 | 1.41 ± 0.02 |

**Supplementary Table 9.** Statistical comparison of folding stability between EquiDesign and baseline methods on 9 representative proteins (mean $\pm$ 95%CI)

| **Comparison** | **Metrics** | **Mean difference** | **95% CI** | **p-value** |
| --- | --- | --- | --- | --- |
| EquiDesign vs. ProteinMPNN | Stability score | +0.3992 | (0.1634, 0.6350) | 4.5153E-03 |
| EquiDesign vs. PiFold | Stability score | +0.7019 | (0.1019, 1.3018) | 2.7167E-02 |
| EquiDesign vs. GeoSeqBuilder | Stability score | +0.3337 | (-0.3284, 0.9958) | 2.7867E-01 |
| EquiDesign vs. ProtSeqGen | Stability score | +0.4990 | (-0.0557, 1.0537) | 7.1749E-02 |

**Supplementary Table 10.** Rosetta Energy Evaluation of EquiDesign-Designed and Native Sequences

| **Protein** | **EquiDesign-predicted sequence** | | | **Native sequence** | | |
| --- | --- | --- | --- | --- | --- | --- |
|  | **Total energy** | **Fa_atr** | **Fa_rep** | **Total energy** | **Fa_atr** | **Fa_rep** |
| 1f89.A | -710.653 | -1582.421 | 199.843 | -695.124 | -1579.357 | 196.945 |
| 1luc.A | -1019.647 | -1991.523 | 231.642 | -989.383 | -2001.044 | 223.562 |
| 1n27.A | -228.413 | -462.167 | 57.497 | -227.457 | -445.282 | 49.157 |
| 1i07.A | -115.501 | -222.406 | 19.099 | -106.540 | -234.530 | 22.663 |
| 1a4i.A | -933.316 | -1644.828 | 177.586 | -923.199 | -1662.903 | 178.011 |
| 1xe7.A | -608.975 | -1045.348 | 119.750 | -586.606 | -1065.706 | 125.582 |
| 3u5t.A | -645.677 | -1243.587 | 142.667 | -673.694 | -1247.327 | 137.928 |
| 2qk2.A | -755.621 | -1384.439 | 157.206 | -724.300 | -1394.437 | 153.872 |
| 3h36.A | -231.879 | -430.684 | 38.402 | -238.456 | -440.442 | 38.701 |

**Supplementary Table 11.** Diversity and foldability analysis of EquiDesign-generated sequences via top-k sampling. Mean values for the first three metrics are reported. Foldability calculates the proportion of the four sequences that satisfy scRMSD < 2.0 Å.

| **Protein** | **Seq identity to native (%)** | **Pairwise diversity** | **AA distribution shift** | **Foldability (%)** |
| --- | --- | --- | --- | --- |
| 1f89.A | 44.834 | 15.560 | 0.066 | 75 |
| 1luc.A | 53.528 | 16.053 | 0.037 | 75 |
| 1n27.A | 46.875 | 17.361 | 0.378 | 75 |
| 1i07.A | 35.169 | 17.232 | 0.371 | 50 |
| 1a4i.A | 63.596 | 15.556 | 0.024 | 100 |
| 1xe7.A | 61.694 | 16.487 | 0.175 | 100 |
| 3u5t.A | 65.217 | 14.348 | 0.299 | 100 |
| 2qk2.A | 58.120 | 12.322 | 0.054 | 100 |
| 3h36.A | 42.949 | 13.034 | 0.206 | 75 |

**Supplementary Table 12.** Run time and memory consumption of pretrained models on TS50 and TS500

| **Dataset** | **Method** | **Runtime (s)** | **Memory (GB)** |
| --- | --- | --- | --- |
| TS50 | ProteinMPNN | 1.787 | 1.316 |
|  | PiFold | 1.535 | 1.751 |
|  | GeoSeqBuilder | 2.307 | 1.513 |
|  | ProtSeqGen | 1.516 | 1.199 |
|  | EquiDesign | 1.235 | 1.193 |
| TS500 | ProteinMPNN | 8.711 | 2.755 |
|  | PiFold | 7.603 | 3.463 |
|  | GeoSeqBuilder | 11.138 | 3.285 |
|  | ProtSeqGen | 7.421 | 2.410 |
|  | EquiDesign | 5.863 | 2.397 |

**Supplementary Table 13.** Statistical comparison on IDRome-120 between EquiDesign and other methods (mean $\pm$ 95%CI)

| **Comparison** | **Metrics** | **Mean difference** | **95% CI** | **p-value** |
| --- | --- | --- | --- | --- |
| EquiDesign vs. ProteinMPNN | Recovery (%) $\boldsymbol{\uparrow}$ | 13.5144 | (13.2746, 13.7454) | 4.8564E-16 |
|  | Perplexity $\boldsymbol{\downarrow}$ | -6.3716 | (-6.5429, -6.1971) | 2.6136E-14 |
|  | Loss $\boldsymbol{\downarrow}$ | -1.5583 | (-1.5804, -1.5396) | 3.6929E-17 |
| EquiDesign vs. PiFold | Recovery (%) $\boldsymbol{\uparrow}$ | 8.6209 | (8.4599, 8.7801) | 8.6263E-16 |
|  | Perplexity $\boldsymbol{\downarrow}$ | -3.5776 | (-3.7274, -3.4326) | 1.1030E-12 |
|  | Loss $\boldsymbol{\downarrow}$ | -1.1462 | (-1.1656, -1.1344) | 5.0190E-17 |
| EquiDesign vs. GeoSeqBuilder | Recovery (%) $\boldsymbol{\uparrow}$ | 7.4763 | (7.2978, 7.6622) | 9.8636E-15 |
|  | Perplexity $\boldsymbol{\downarrow}$ | -2.5181 | (-2.6454, -2.3946) | 6.0165E-12 |
|  | Loss $\boldsymbol{\downarrow}$ | -1.0012 | (-1.0165, -0.9835) | 2.9474E-16 |
| EquiDesign vs. ProtSeqGen | Recovery (%) $\boldsymbol{\uparrow}$ | 3.5814 | (3.429, 3.731) | 1.3673E-12 |
|  | Perplexity $\boldsymbol{\downarrow}$ | -1.1977 | (-1.2733, -1.1267) | 3.7677E-11 |
|  | Loss $\boldsymbol{\downarrow}$ | -0.0892 | (-0.1006, -0.0794) | 1.3301E-08 |

**Supplementary Table 14.** Statistical comparison on TS45 between EquiDesign and other methods (mean $\pm$ 95%CI)

| **Comparison** | **Metrics** | **Mean difference** | **95% CI** | **p-value** |
| --- | --- | --- | --- | --- |
| EquiDesign vs. ProteinMPNN | Recovery (%) $\boldsymbol{\uparrow}$ | 11.0941 | (10.8684, 11.3116) | 1.6613E-15 |
|  | Perplexity $\boldsymbol{\downarrow}$ | -3.9345 | (-4.0645, -3.7955) | 2.0935E-13 |
|  | Loss $\boldsymbol{\downarrow}$ | -0.6324 | (-0.6631, -0.5969) | 9.8514E-12 |
| EquiDesign vs. PiFold | Recovery (%) $\boldsymbol{\uparrow}$ | 5.3406 | (5.2524, 5.4276) | 2.8289E-16 |
|  | Perplexity $\boldsymbol{\downarrow}$ | -2.0162 | (-2.1004, -1.8996) | 6.5089E-12 |
|  | Loss $\boldsymbol{\downarrow}$ | -0.1286 | (-0.1428, -0.1172) | 2.7398E-09 |
| EquiDesign vs. GeoSeqBuilder | Recovery (%) $\boldsymbol{\uparrow}$ | 4.8617 | (4.6844, 5.0356) | 3.4089E-13 |
|  | Perplexity $\boldsymbol{\downarrow}$ | -1.7468 | (-1.8522, -1.6478) | 2.5209E-11 |
|  | Loss $\boldsymbol{\downarrow}$ | -0.1495 | (-0.1703, -0.1297) | 4.3439E-08 |
| EquiDesign vs. ProtSeqGen | Recovery (%) $\boldsymbol{\uparrow}$ | 3.3479 | (3.2556, 3.4444) | 3.6620E-14 |
|  | Perplexity $\boldsymbol{\downarrow}$ | -0.6758 | (-0.7448, -0.6152) | 1.9985E-09 |
|  | Loss $\boldsymbol{\downarrow}$ | -0.1074 | (-0.1269, -0.0931) | 1.3338E-07 |

**Supplementary Table 15.** Stability comparison on CATH 4.2 and CATH 4.4

| **Stability score** | **CATH 4.2** | **CATH 4.4** |
| --- | --- | --- |
| ProteinMPNN | 8.118$\pm$2.471 | 8.446$\pm$2.108 |
| PiFold | 8.212$\pm$2.689 | 8.338$\pm$2.326 |
| GeoSeqBuilder | 8.276$\pm$2.353 | 8.458$\pm$2.571 |
| ProtSeqGen | 8.349$\pm$2.486 | 8.575$\pm$2.213 |
| EquiDesign | 8.421$\pm$2.293 | 8.648$\pm$2.138 |

**Supplementary Table 16.** Confidence of EquiDesign-predicted and scrambled sequences against native sequences for 9 representative proteins. RMSD quantifies the structural deviation of the AF3-predicted design from the native backbone. ‘Foldable’ is equal to ‘Yes’ if RMSD < 2.0Å.

| **Protein** | **EquiDesign-predicted sequence** | | | **Scrambled sequence** | | |
| --- | --- | --- | --- | --- | --- | --- |
|  | pLDDT | RMSD (Å) | Foldable | pLDDT | RMSD (Å) | Foldable |
| 1f89.A | 88.86 | 1.49 | Yes | 25.63 | 22.52 | No |
| 1luc.A | 92.25 | 0.41 | Yes | 26.13 | 24.48 | No |
| 1n27.A | 83.59 | 1.46 | Yes | 35.92 | 16.98 | No |
| 1i07.A | 86.11 | 0.86 | Yes | 53.47 | 13.17 | No |
| 1a4i.A | 95.32 | 0.38 | Yes | 26.93 | 22.68 | No |
| 1xe7.A | 95.26 | 1.13 | Yes | 34.22 | 22.97 | No |
| 3u5t.A | 95.11 | 0.64 | Yes | 34.13 | 22.22 | No |
| 2qk2.A | 89.16 | 1.40 | Yes | 41.59 | 22.04 | No |
| 3h36.A | 86.34 | 0.36 | Yes | 78.62 | 33.91 | No |

**Supplementary Table 17.** Sequence conservation and structural neighbor analysis of representative proteins using MMseqs2 and Foldseek

| **Protein** | **Sequence identity search** | | **Nearest structural neighbor** | |
| --- | --- | --- | --- | --- |
|  | Matched proteins | Max Seq identity (%) | Matched neighbors | Max TM-score |
| 1f89.A | 1f89.A; 1f89.B | 96.441 | 1f89.B | 1.00 |
| 1luc.A | 1luc.A | 91.831 | 1luc.A | 1.00 |
| 1n27.A | 1n27.A | 100 | 1n27.A | 1.00 |
| 1i07.A | 1i07.A; 1i07.B | 100 | 1i07.B | 0.926 |
| 1a4i.A | 1a4i.A; 1a4i.B | 96.610 | 1a4i.A | 1.00 |
| 1xe7.A | 1xe7.A; 1xe7.B; 1xe7.C | 95.876 | 1xe7.A | 1.00 |
| 3u5t.A | 3u5t.A; 3u5t.B; 3u5t.C; 3u5t.D | 95.436 | 3u5t.A | 1.00 |
| 2qk2.A | 2qk2.A | 100 | 2qk2.A | 1.00 |
| 3h36.A | 3h36.A | 100 | 3h36.A | 1.00 |
